# Asymmetric Fluorinated Spiro Hole‐Transport Materials Enabled by a Rapid Core‐Forming Strategy for Blade‐Coated Perovskite Solar Cells

**DOI:** 10.1002/advs.77623

**Published:** 2026-09-08

**Authors:** Kun‐Mu Lee, Xiu‐Ping Lin, Ya‐Ho Chang, Cheng‐Ting Tsai, Pei‐Chen Chu, Yu‐Hsun Chen, Wei‐Hao Chiu, Yu‐Che Lin, Sie‐Rong Li, Hsiao‐Chi Hsieh, Kang‐Ling Liau, Shih‐I. Lu, Chih‐Min Wang, Yan‐Duo Lin

**Affiliations:** ^1^ Department of Chemical and Materials Engineering Chang Gung University Taoyuan Taiwan; ^2^ Center For Sustainability and Energy Technologies Chang Gung University Taoyuan Taiwan; ^3^ Division of Neonatology Department of Pediatrics Chang Gung Memorial Hospital Linkou Taoyuan Taiwan; ^4^ College of Environment and Resources Ming Chi University of Technology New Taipei City Taiwan; ^5^ Department of Chemistry Soochow University Taipei Taiwan; ^6^ Department of Bioscience and Biotechnology National Taiwan Ocean University Keelung Taiwan; ^7^ Institute of Chemistry Academia Sinica Taipei Taiwan; ^8^ Department of Chemical and Materials Engineering Tamkang University New Taipei City Taiwan; ^9^ Department of Chemistry National Central University Taoyuan Taiwan

**Keywords:** blade‐coating fabrication, cyclopenta[2,1‐b;3,4‐b’]dithiophene, hole‐transporting material, long‐term stability, Perovskite solar cell, spiro‐type structure

## Abstract

Developing hole‐transporting materials (HTMs) that simultaneously enable high efficiency, stability, and scalable processing remains a key challenge for perovskite solar cells (PSCs). Here, we report the facile synthesis of asymmetric fluorinated spiro‐type HTMs that integrate molecular design with scalable fabrication. The controlled incorporation and positioning of fluorine within a common asymmetric molecular framework tune the energy levels, solid‐state organization, and interfacial interactions of the HTMs. Coupling these materials with a fully blade‐coating–based fabrication process and vacuum‐assisted crystallization enables controlled film formation, yielding uniform, compact films with reduced defect density. As a result, PSCs based on the optimized HTM achieve a PCE of 24.49%, maintaining 23.28% for large‐area (1.00 cm^2^) devices with excellent operational stability. This work establishes a synergistic molecular–processing engineering strategy toward high‐performance and manufacturable PSCs.

## Graphical abstract

Facile synthesis of asymmetric fluorinated spiro‐type HTMs combined with blade‐coating fabrication and vacuum‐assisted crystallization enables high‐efficiency PSCs with PCEs of 24.49% (0.09 cm^2^) and 23.28% (1.00 cm^2^), along with excellent stability.

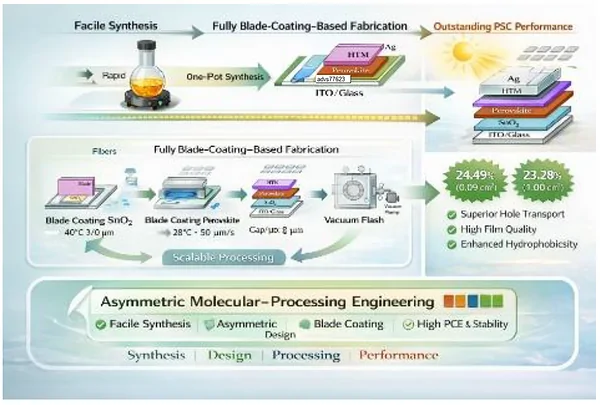

## 1 Introduction

Perovskite solar cells (PSCs) have rapidly emerged as one of the most promising photovoltaic technologies for efficient and cost‐effective solar energy conversion. Since the pioneering work of Miyasaka and co‐workers in 2009, the field has witnessed remarkable progress with power conversion efficiencies (PCEs) soaring to values as high as 28% within just over a decade of research, comparable to the performance of established monocrystalline silicon solar cells [1]. This exceptional rise stems from the unique optoelectronic properties of halide perovskites, including their large absorption coefficients, tunable bandgaps, long carrier diffusion lengths, and efficient ambipolar charge transport [2, 3, 4, 5, 6]. In PSCs, the hole‐transporting material (HTM) plays a pivotal role not only as a charge‐selective contact facilitating efficient hole extraction and transport but also as a protective interlayer that shields the perovskite absorber from deleterious ambient conditions such as moisture [7, 8]. Over the past decade, extensive efforts have been devoted to the chemical design and optimization of HTMs, encompassing inorganic compounds [10], polymeric structures [11, 12], and organic small molecules [9, 13, 14]. Among these, small organic molecules have attracted particular attention due to their structural versatility, relatively straightforward synthesis and purification, and the possibility of fine‐tuning their optoelectronic properties through rational molecular engineering. The prototypical small‐molecule HTM, spiro‐OMeTAD (2,2′,7,7′‐tetrakis(*N*,*N*‐di‐4‐methoxyphenylamino)‐9,9′‐spirobifluorene), has been widely adopted in high‐efficiency PSCs owing to its ability to form uniform thin films and promote efficient charge transfer, thereby minimizing recombination losses [15, 16, 17]. Despite its success, spiro‐OMeTAD presents serious challenges that hinder its practical deployment. Its synthesis requires multiple low‐temperature steps involving air‐ and moisture‐sensitive reagents such as n‐butyllithium and Grignard reagents as well as highly reactive halogenating agents, ultimately affording yields below 30%. Moreover, sublimation‐grade, high‐purity spiro‐OMeTAD is required to ensure optimal device performance, further exacerbating production costs. Other limitations, including low intrinsic charge‐carrier mobility and poor thermal and environmental stability, significantly restrict its long‐term operational stability and scalability. These drawbacks highlight the urgent need for alternative HTMs that can simultaneously deliver efficient charge transport, robust stability, facile synthesis, and compatibility with scalable fabrication processes. Recent reports on spiro‐based derivatives, heterocyclic and heteropolycyclic aromatic units, and donor–acceptor‐type systems have demonstrated promising charge‐transport characteristics and improved stability profiles [18, 19, 20, 21, 22, 23]. These emerging candidates not only address the synthetic and economic limitations of spiro‐OMeTAD but also open new pathways toward the realization of cost‐effective, stable, and highly efficient PSCs suitable for large‐scale applications.

Cyclopenta[2,1‐b:3,4‐b’]dithiophene (CPDT) is a versatile building unit for organic optoelectronic materials owing to its rigid π‐conjugated framework and excellent charge‐transport properties. CPDT‐based derivatives have been widely applied in organic light‐emitting diodes (OLEDs), organic field‐effect transistors (OFETs), and organic photovoltaic devices (OPVs) [24, 25, 26], and, more recently, have emerged as promising HTMs for PSCs, delivering competitive device performance [27, 28, 29, 30, 31, 32, 33, 34, 35, 36, 37]. In particular, spiro‐CPDT architectures have attracted considerable attention, as their three‐dimensional configuration effectively suppresses excessive aggregation while maintaining good film‐forming properties. Early examples, such as spiro‐CPDT derivatives and **FDT**‐based HTMs, have demonstrated progressively improved efficiencies. However, their practical application remains limited by multistep synthetic routes and purification challenges [38, 39, 40].

Among the diverse strategies employed in the design of HTMs, the deliberate incorporation of asymmetric subunits has emerged as a particularly effective approach. Recent studies have consistently demonstrated that molecular asymmetry not only enhances intermolecular interactions and polarization but also fine‐tunes the energy levels of the resulting materials [20, 41, 42, 43]. Such structural modifications provide a powerful means of optimizing charge transport, stability, and interfacial compatibility, thereby offering significant advantages for next‐generation PSCs. In addition, the incorporation of fluorine atoms into HTMs has emerged as a highly effective molecular engineering strategy. This modification has been shown to significantly strengthen interfacial interactions with perovskite layers, elevate the glass transition temperature (T_g_), and lower the HOMO energy level. In turn, these changes enable more precise energy level alignment, facilitate charge transport, and enhance hydrophobicity, thereby contributing to both improved device efficiency and long‐term operational stability [18, 34, 35, 36, 44]. One effective approach to enhancing the conductivity of hole‐transporting layers lies in the deliberate incorporation of extended π‐conjugated frameworks within the molecular structure of the HTMs. In 2022, Yang and co‐workers reported the development of **Spiro‐Naph**, a spiro‐based HTM bearing asymmetric phenylnaphthylamine end groups, synthesized through a symmetry‐tuning molecular design strategy [20]. The naphthyl unit has a more extended π‐conjugated framework than its phenyl counterpart, markedly enhancing the intrinsic properties of the HTM. This structural modification results in a deeper HOMO energy level, increased hydrophobicity, more compact and stable molecular packing, and superior hole mobility. As a consequence, PSCs incorporating **Spiro‐Naph** not only achieved a remarkable PCE of 24.43% but also exhibited excellent operational stability.

Despite these advances, two critical challenges remain insufficiently addressed. First, many high‐performance HTMs still rely on synthetically demanding, multistep procedures, which limit their scalability and practical implementation. Second, most studies focus primarily on molecular design while overlooking the crucial role of device fabrication processes, particularly for large‐area and industrial‐scale production. Conventional spin‐coating techniques suffer from poor material utilization and limited scalability, making them unsuitable for practical manufacturing. In contrast, blade coating offers a promising alternative due to its compatibility with continuous processing and precise control over film formation. However, achieving uniform, defect‐minimized films via blade coating requires careful regulation of solvent evaporation and crystallization dynamics.

**FIGURE 1:**
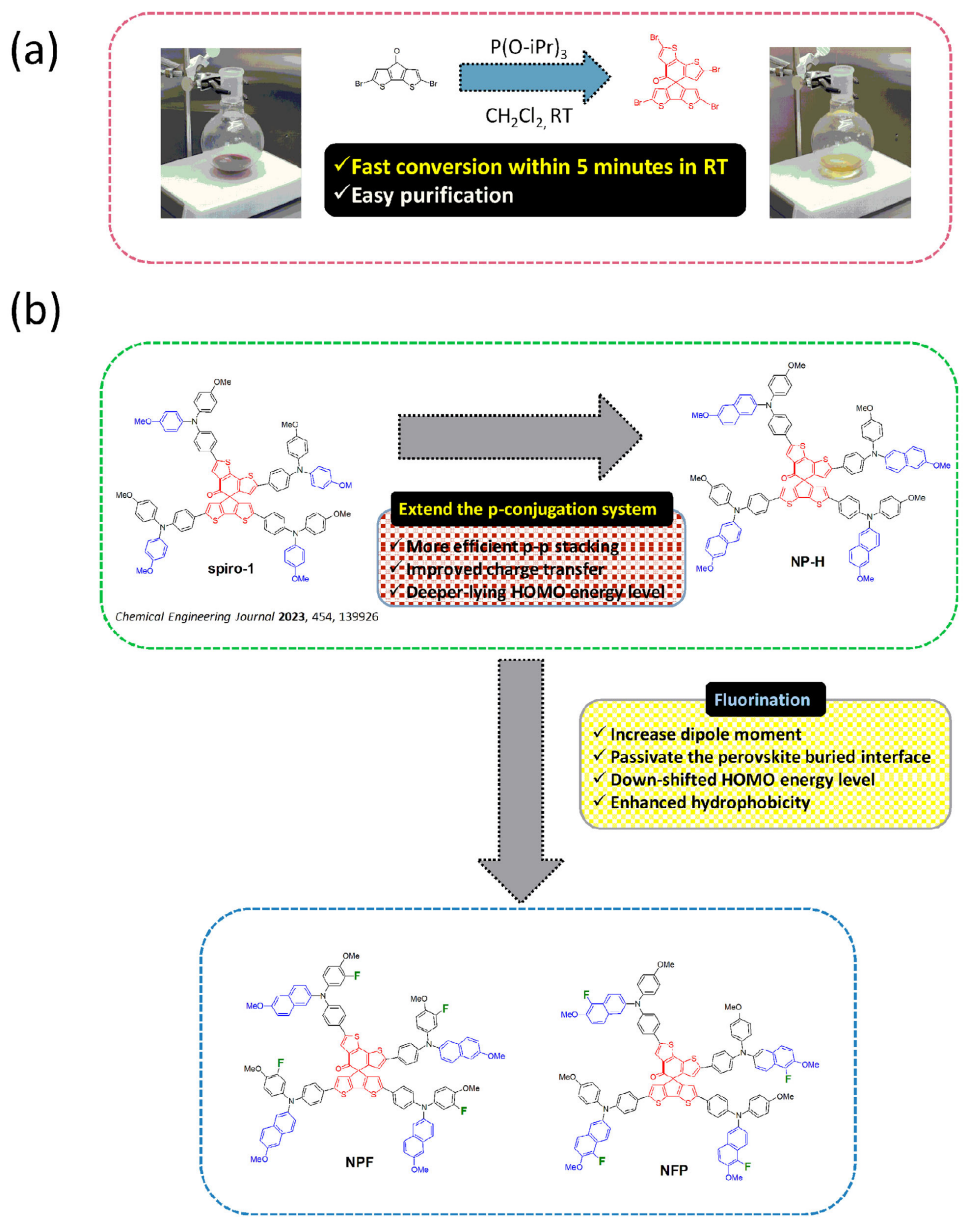
(a) Synthetic routes to the spiro‐CPDT molecular core. (b) Chemical structures of **NPH**, **NPF**, and **NFP**. The spiro‐1 structure and the core‐forming reaction were independently redrawn for this work from chemical information reported in the authors’ previous study [33].

To address these challenges, it is highly desirable to develop an integrated strategy that simultaneously considers synthetic accessibility, molecular design, and scalable processing. In this context, we propose a synergistic molecular–processing engineering approach, in which the intrinsic properties of HTMs and the extrinsic film‐formation processes are co‐optimized to maximize device performance and manufacturability. The previously reported **Spiro‐Naph** study established asymmetric phenylnaphthylamine substitution as an effective strategy for regulating the molecular packing, electronic properties, hole transport, and device stability of spirobifluorene‐based HTMs. Nevertheless, its molecular construction relies on a comparatively elaborate synthetic route, and the effects of fluorination within a sulfur‐rich spiro‐CPDT framework remain insufficiently understood. In the present work, we develop **NPH**, **NPF**, and **NFP** based on a distinct spiro‐CPDT platform whose central spiro framework can be rapidly constructed through a one‐pot transformation within 5 min at room temperature under ambient conditions, followed by simple crystallization purification (Figure 1). The rapid spiro‐CPDT core‐forming reaction employed here was established in our previous study of **spiro‐1** and **spiro‐2** [33]. In those materials, the spiro‐CPDT core was functionalized with four structurally symmetric triarylamine donor arms through phenylene‐ or thiophene‐containing linkers. In contrast, **NPH**, **NPF**, and **NFP** incorporate asymmetric phenylnaphthylamine terminal units with an extended naphthyl π‐system. **NPH** provides the non‐fluorinated reference, whereas **NPF** and **NFP** are positional fluorination isomers, allowing the influence of fluorine incorporation and substitution position to be examined within a common molecular framework. This synthetically accessible platform is combined with asymmetric phenylnaphthyl terminal groups and controlled fluorination to systematically elucidate the effects of fluorine substitution and substitution position on molecular packing, energy‐level alignment, charge transport, interfacial interactions, and photovoltaic performance. Furthermore, these HTMs are integrated with fully blade‐coated functional layers and vacuum‐assisted crystallization, thereby bridging facile molecular synthesis with scalable device manufacturing. As a result, PSCs employing the optimized HTM (**NFP**) achieve a high PCE of 24.49%, while maintaining excellent performance in large‐area (1.00 cm^2^) devices and exhibiting superior environmental, thermal, and operational stability. This work establishes a general framework that bridges facile synthesis, molecular engineering, and scalable device fabrication, offering a viable pathway toward the practical deployment of perovskite solar cells.

## 2 Results and Discussion

The synthetic procedures for **NPH**, **NPF**, and **NFP** are outlined in Scheme S1. The chemical structures of all products were unambiguously confirmed by comprehensive spectroscopic characterization, including ^1^H, ^13^C, and ^19^F NMR spectroscopy as well as high‐resolution mass spectrometry (Figures S1–S44). Building on our previously established rapid spiro‐CPDT core‐forming reaction, the present work expands this synthetic platform to previously unreported asymmetric phenylnaphthylamine‐containing HTMs with controlled fluorination. This facile and scalable synthetic route provides a promising platform for practical HTM development. Notably, purification at this stage was achieved by simple crystallization prior to the subsequent transformation. Moreover, we successfully obtained single crystals of the key spiro intermediate **3** (CCDC 2174353, Figure S45), which revealed that the two fused heteroaromatic moieties adopt an almost orthogonal orientation about the central tetrahedral carbon [33]. Ultimately, the desired small molecules **NPH**, **NPF**, and **NFP** were synthesized in high yields via Suzuki cross‐coupling reactions.

Thermogravimetric analysis (TGA) and differential scanning calorimetry (DSC) were employed to elucidate the thermal characteristics of the newly developed HTMs. As depicted in Figure S46, all HTMs exhibit excellent thermal stability with decomposition temperatures (T_d_, defined as the temperature corresponding to 5% weight loss) exceeding 410°C (Table 1), indicating their suitability for device fabrication under thermal stress conditions. Notably, the fluorinated derivatives display slightly enhanced stability, with T_d_ values of 418°C for **NPF** and 430°C for **NFP**, compared to 413°C for their non‐fluorinated analogue **NPH**. The DSC thermograms (Figure S47) demonstrate that the HTMs **NPH**, **NPF**, and **NFP** exhibit comparable morphological characteristics, with glass transition temperatures (T_g_) of 109, 111, and 114°C, respectively. Importantly, the fluorinated derivatives (**NPF** and **NFP**) show slightly elevated glass transition temperatures compared to **NPH**, suggesting enhanced molecular rigidity and stronger intermolecular interactions. These higher Tg values are beneficial for maintaining morphological stability during device operation. Powder X‐ray diffraction (PXRD) confirms the predominantly amorphous nature of the films, which is critical for forming uniform and pinhole‐free thin films (Figure S48). The combination of amorphous morphology and enhanced thermal robustness ensures stable film formation and long‐term device durability.

**TABLE 1:** Photophysical, electrochemical, and thermal data of **NPH**, **NPF**, and **NFP**.

HTM	*λ*_abs_ (nm) (*ε*× 10^−4^/M^−1^ cm^−1^) ^a^	*λ*_f_ (nm) ^a^	*E*_HOMO_ (eV) ^b^	*E*_0‐0_ (eV) ^c^	*E*_LUMO_ (eV) ^d^	*E*_HOMO_ (eV) ^e^	*E*_0‐0_ (eV) ^e^	*E*_LUMO_ (eV) ^e^	T*_g_* (°C)^f^	T*_d_* (°C)^f^
**NPH**	449 (12.5)	559	−5.24	2.49	−2.75	−4.28	2.40	−1.88	109	413
**NPF**	448 (15.1)	520	−5.29	2.52	−2.77	−4.41	2.43	−1.98	111	418
**NFP**	449 (14.9)	519	−5.33	2.52	−2.81	−4.48	2.50	−1.98	114	430

^a^ Maxima of the absorption and fluorescence bands in THF solution.

^b^ Determined by differential pulse voltammetry (DPV).

^c^ The value of *E*_0‐0_ was obtained from the intersection of normalized absorption and photoluminescence spectra.

^d^ The LUMO energies of the compounds were estimated as *E*_HOMO_ + *E*_0‐0_.

^e^ Values were calculated at the B3LYP/6‐31G(d,p) level.

^f^ Glass transition (T_g_) and decomposition (T_d_) temperatures determined by DSC and TGA, respectively.

**FIGURE 2:**
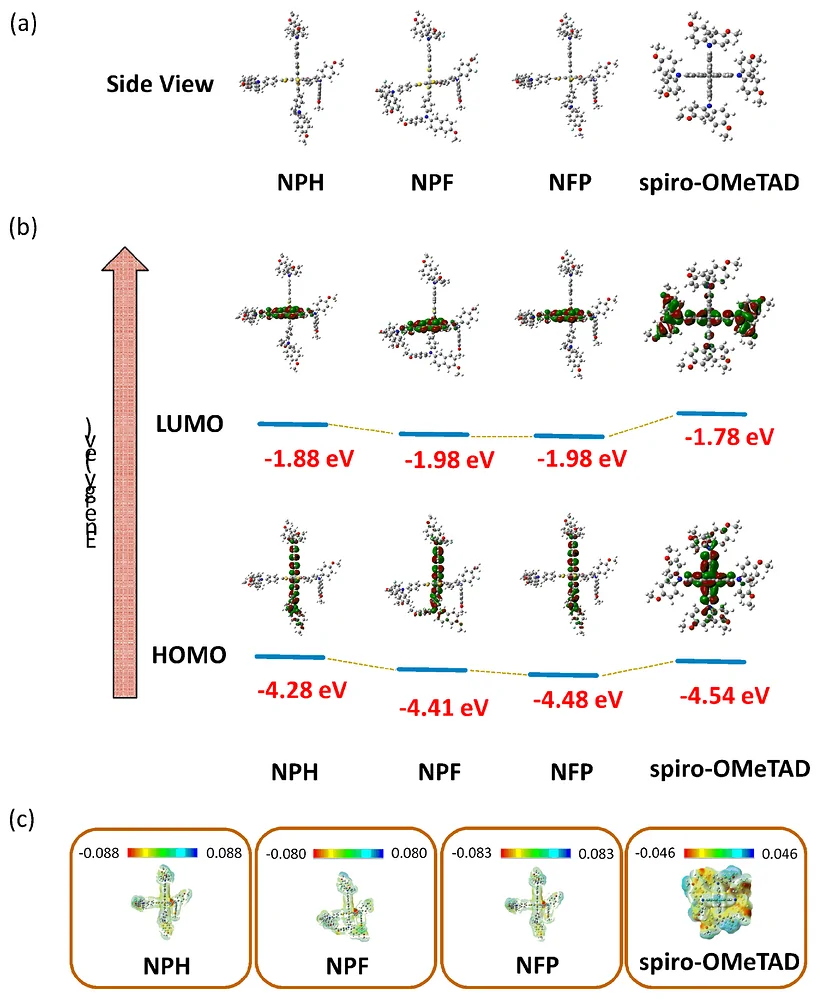
(a) Side views of the structures of **NPH**, **NPF**, **NFP**, and spiro‐OMeTAD optimized using DFT at the B3LYP/6‐31G(d,p) level. (b) Spatial distributions of the HOMOs and LUMOs obtained from DFT calculations. (c) DFT‐calculated ESP surfaces of **NPH**, **NPF**, **NFP**, and spiro‐OMeTAD.

To gain a deeper understanding of the spatial conformations and electronic characteristics of **NPH**, **NPF**, and **NFP**, we conducted density functional theory (DFT) calculations at the B3LYP/6‐31G(d,p) level. For comparison, spiro‐OMeTAD was also included in the analysis (Figure 2). The results reveal that the CPDT core and the benzodithiophenone fragment adopt nearly orthogonal orientations that are consistent with single‐crystal X‐ray diffraction analysis (Figure S45) [33] and resemble the structural configuration observed for spiro‐OMeTAD. As illustrated in Figure 2, the highest occupied molecular orbitals (HOMOs) of **NPH**, **NPF**, and **NFP** are predominantly delocalized over the arylamine donor and CPDT units, whereas the lowest unoccupied molecular orbitals (LUMOs) are largely localized on the benzodithiophenone segment and the adjacent phenyl substituents. The calculated HOMO energy levels are −4.28, −4.41, and −4.48 eV for **NPH**, **NPF**, and **NFP**, respectively, while the corresponding LUMO energies are −1.88, −1.98, and −1.98 eV. These values yield HOMO–LUMO energy gaps (E_g_) of 2.40, 2.43, and 2.50 eV, respectively. Importantly, fluorination induces a systematic downshift of both HOMO and LUMO energy levels, leading to improved alignment with the valence band of the perovskite layers. Such optimized energy‐level alignment reduces energetic losses during hole extraction and contributes to enhanced open‐circuit voltage (*V*_oc_). Figure 2c illustrates the molecular electrostatic potential (ESP) surfaces derived from DFT calculations. The ESP analysis reveals that the negative potential of **NPH**, **NPF**, and **NFP** (highlighted in red) is predominantly localized on the benzodithiophenone moiety. This distribution implies that, upon deposition onto a perovskite film, these molecules are expected to exhibit stronger interactions with positively charged defect states, particularly the undercoordinated Pb^2^^+^ centers, thereby contributing to effective defect passivation.

**FIGURE 3:**
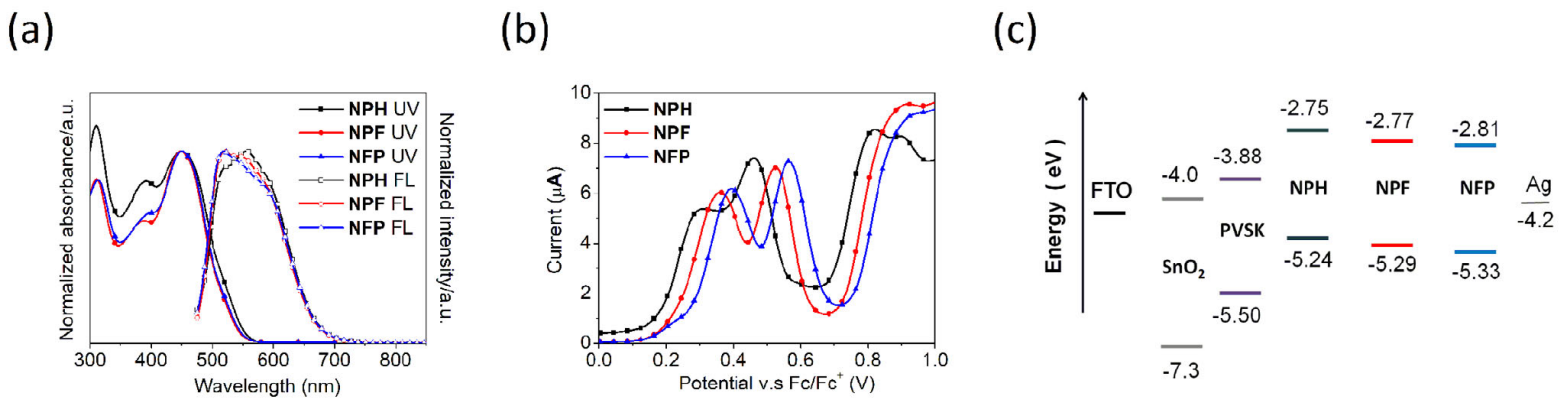
(a) Normalized absorption and photoluminescence spectra of **NPH**, **NPF**, and **NFP** in THF solution. (b) Differential pulse voltammetry (DPV) curves of **NPH**, **NPF**, and **NFP**. (c) Energy‐level alignment diagram of the HTMs used in the investigated devices.

The normalized UV–vis absorption and photoluminescence (PL) spectra of **NPH**, **NPF**, and **NFP** in THF are presented in Figure 3a, with the corresponding photophysical parameters summarized in Table 1. All three HTMs exhibit two similar absorption bands in the 300–310 nm and 425–500 nm regions, consistent with their common backbone structure. The intense high‐energy absorption features are assigned to π–π* transitions of the conjugated backbone, while the lower‐energy bands arise from intramolecular charge‐transfer (ICT) transitions from donor to acceptor units, consistent with the predictions of DFT calculations. Furthermore, time‐dependent density functional theory (TDDFT) calculations were performed to simulate the absorption spectra of **NPH**, **NPF**, and **NFP**. Despite differences in λ_max_, the experimental trends agreed well with the calculated results (Figure S49). The PL spectra of **NPH**, **NPF**, and **NFP** reveal emission maxima at 559, 520, and 519 nm, respectively. The intersection points of the normalized absorption and PL spectra for **NPH**, **NPF**, and **NFP** are 497, 493, and 492 nm, corresponding to E_0–0_ values of 2.49, 2.52, and 2.52 eV, respectively. The fluorinated derivatives display slightly blue‐shifted emission and modified optical band gaps, reflecting the influence of electron‐withdrawing substituents on the electronic structure. Electrochemical measurements further confirm the gradual stabilization of the HOMO levels upon fluorination.

We determined the HOMO energy levels of the target molecules by cyclic voltammetry (CV, Figure S50) and differential pulse voltammetry (DPV, Figure 3b) in THF solution, using ferrocene/ferrocenium (Fc/Fc^+^) as the internal reference. The corresponding energy level diagram for **NPH**, **NPF**, and **NFP** is depicted in Figure 3c. Analysis of the DPV traces revealed HOMO values of −5.24, −5.29, and −5.33 eV for **NPH**, **NPF**, and **NFP**, respectively, which are slightly higher in energy than the valence band maximum of the perovskite layer (−5.50 eV), thereby ensuring efficient hole transfer from the perovskite absorber to the HTM layer at the interface. Notably, the incorporation of fluorine substituents leads to a progressive stabilization of the HOMO level, yielding values that are optimally aligned with the perovskite valence band. Such alignment reduces energetic losses during hole extraction and contributes to enhanced *V*_oc_. The LUMO levels of **NPH**, **NPF**, and **NFP** were derived by combining their HOMO levels with their optical band gaps, giving values of −2.75, −2.77, and −2.81 eV, respectively. All three levels lie above the conduction band minimum of the perovskite, which is advantageous for suppressing parasitic electron back‐transfer from the perovskite to the metallic electrode through the HTM. Moreover, the experimental HOMO and LUMO levels determined from DPV exhibit a consistent trend with those predicted by DFT calculations, reinforcing the reliability of the measurements. These results collectively demonstrate that fluorination provides a powerful strategy to fine‐tune the electronic properties of HTMs, enabling improved charge extraction and reduced recombination losses in PSCs.

**FIGURE 4:**
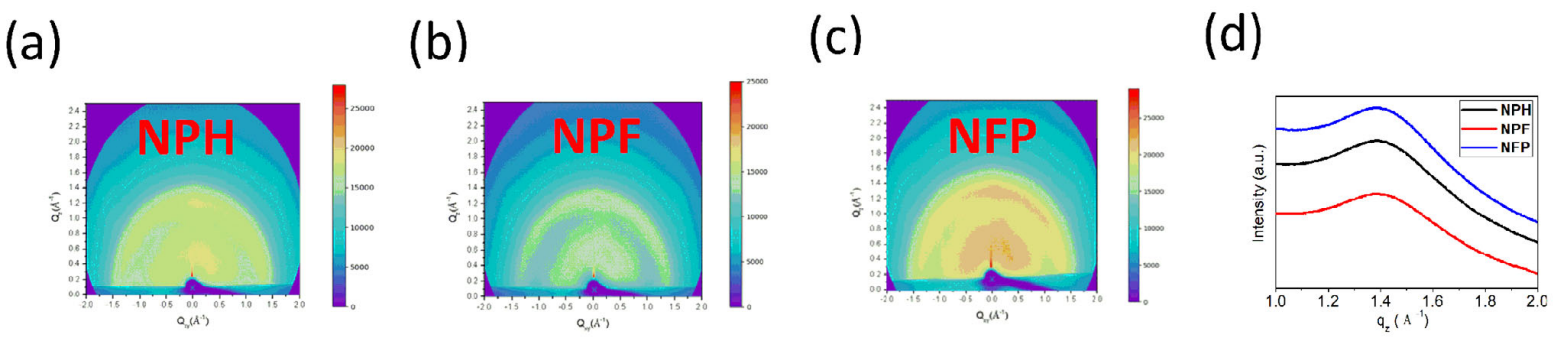
Two‐dimensional GIWAXS patterns of (a) **NPH** film, (b) **NPF** film, (c) **NFP** film, and (d) out‐of‐plane line cuts of **NPH**, **NPF**, and **NFP**.

To elucidate the intrinsic charge‐transport characteristics, space‐charge‐limited current (SCLC) measurements were conducted to determine the hole mobilities of **NPH**, **NPF**, and **NFP** using hole‐only devices with the configuration ITO/PEDOT:PSS/HTM/Al, with spiro‐OMeTAD serving as a reference (Figure S51) under identical experimental conditions. Thus, the obtained mobilities allow a reliable comparison of the relative charge‐transport capabilities of the different molecular structures. It should be noted that the SCLC measurements were performed using doped HTM films prepared by spin coating. The extracted hole mobilities for **NPH**, **NPF**, and **NFP** are 1.94 × 10^−^^4^, 3.81 × 10^−^^4^, and 4.21 × 10^−^^4^ cm^2^ V^−^^1^ s^−^^1^, respectively. Notably, the fluorinated HTMs display slightly enhanced hole mobilities relative to their non‐fluorinated counterpart and to the benchmark spiro‐OMeTAD (2.70 × 10^−^^4^ cm^2^ V^−^^1^ s^−^^1^), indicating more efficient hole transport. This improvement can be rationalized by the presence of strong dipole–dipole interactions and tighter molecular packing imparted by the donor–acceptor (D–A) architecture, which collectively facilitate charge transfer [45]. To further elucidate the influence of fluorine substitution on molecular packing and film orientation, grazing‐incidence wide‐angle X‐ray scattering (GIWAXS) measurements were performed. As illustrated in Figure 4, the **NPH**, **NPF**, and **NFP** films exhibit distinct out‐of‐plane π–π stacking diffraction peaks centered at 1.47, 1.48, and 1.49 Å^−^^1^, respectively, indicating a predominant face‐on molecular orientation that is favorable for vertical charge transport in PSCs. The corresponding π–π stacking distances were calculated to be 4.25, 4.23, and 4.22 Å for **NPH**, **NPF**, and **NFP**, respectively, suggesting only a slight reduction in intermolecular spacing upon fluorination. To further quantify the degree of molecular ordering, the out‐of‐plane π–π stacking peaks were fitted with Gaussian functions, and the coherence lengths were estimated from the full width at half maximum (FWHM) according to L = 0.9 × 2π/Δq. The calculated coherence lengths are 1.45, 1.48, and 1.47 nm for **NPH**, **NPF**, and **NFP**, respectively. The comparable coherence lengths indicate that fluorination and its substitution position have only a minor influence on the size of the coherently ordered domains. Therefore, the enhanced hole transport observed for the fluorinated HTMs is unlikely to originate from a significant increase in crystalline domain size. Instead, the improved charge‐transport characteristics are more reasonably attributed to subtle optimization of molecular orientation, intermolecular electronic coupling, and local packing quality induced by controlled fluorine incorporation within the common asymmetric molecular framework.

**FIGURE 5:**
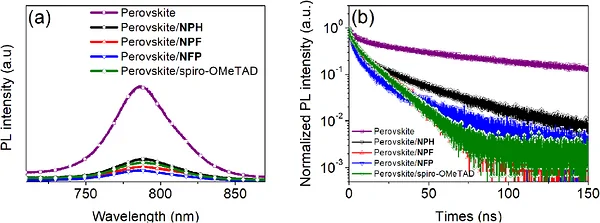
(a) Steady–state photoluminescence spectra and (b) time‐resolved photoluminescence (TRPL) spectra of a pristine perovskite film and perovskite films coated with different HTMs.

To gain deeper insight into the charge extraction dynamics at the perovskite/HTM interface, we conducted steady‐state photoluminescence (PL) and time‐resolved PL (TRPL) measurements on perovskite films with and without HTM layers, as shown in Figure 5a,b. Upon deposition of HTMs, a pronounced quenching of the PL signal was observed, reflecting efficient interfacial charge transfer. The quenching efficiencies follow the order **NFP** > **NPF** > spiro‐OMeTAD > **NPH**, which correlates well with the measured hole mobility values. Notably, fluorinated HTMs exhibited substantially stronger quenching behavior than their non‐fluorinated counterpart, underscoring their superior ability to promote hole extraction. In particular, the perovskite/**NPF** and perovskite/**NFP** interfaces demonstrated more effective PL suppression compared with the perovskite/**NPH** interface, highlighting the critical role of fluorination in enhancing interfacial charge‐transfer processes. In addition, the TRPL decay profiles were analyzed using a biexponential fitting model to extract the radiative lifetimes of the corresponding samples. The detailed fitting parameters are summarized in Table S1. The average PL lifetimes (τ_ave_) of the films coated with **NPH**, **NPF**, **NFP**, and spiro‐OMeTAD were determined to be 13.72, 9.28, 6.93, and 9.67 ns, respectively, exhibiting a consistent trend with the steady‐state PL intensities. Notably, the two fluorinated HTMs (**NPF** and **NFP**) exhibited more pronounced PL quenching along with shorter PL decay times. This behavior is plausibly associated with stronger molecular packing, likely facilitated by dipole–dipole interactions between the fluorinated substituents.

**FIGURE 6:**
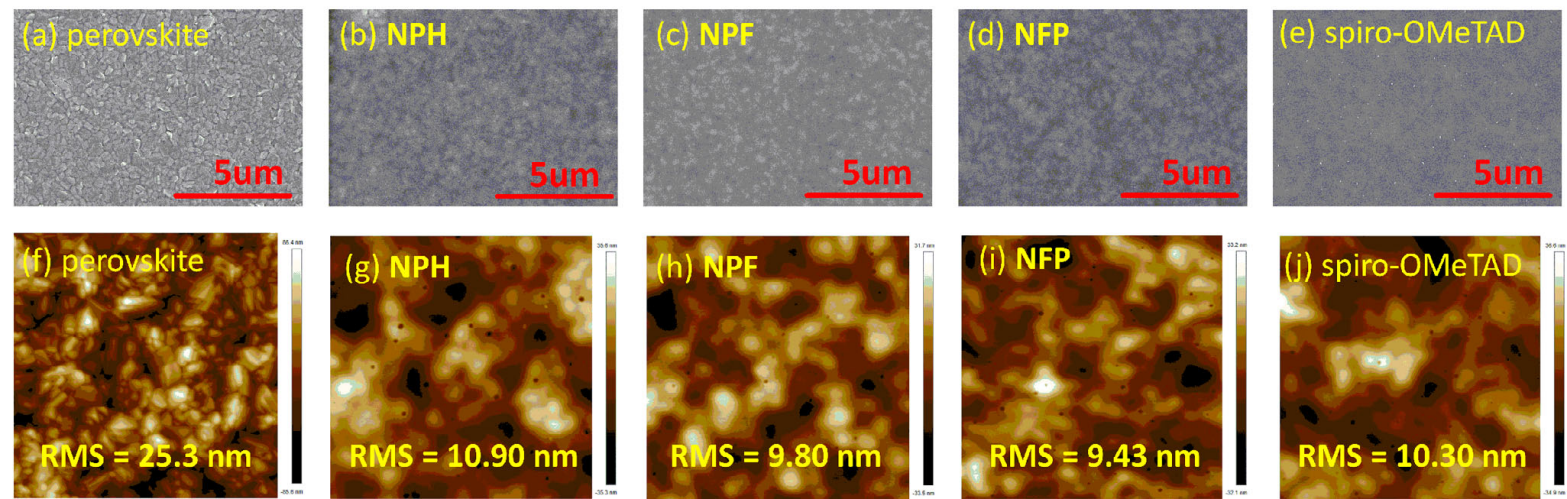
Top‐view SEM images (a–e) and AFM images (f–j) of a pristine perovskite film and perovskite films coated with the HTMs.

A uniform film morphology plays a pivotal role in facilitating efficient charge carrier collection and transport as well as ensuring the long‐term stability of PSCs. To gain deeper insight into the morphological characteristics of the HTM layers on perovskite films, we employed scanning electron microscopy (SEM) and atomic force microscopy (AFM) analyses. Comparative SEM images of perovskite films (Figure 6a–e), with and without HTM coverage, confirm that the **NPH**, **NPF**, and **NFP** layers provide uniform and pinhole‐free coverage on the perovskite film. In addition, AFM analyses (Figure 6f–j) further demonstrate that the fluorinated HTMs, **NPF** and **NFP**, afford smooth and homogeneous films with root‐mean‐square (RMS) roughness values of 9.80 and 9.43 nm, respectively. By contrast, the non‐fluorinated analogue **NPH** exhibits a rougher surface, with an RMS roughness of 10.90 nm. Such smooth and uniform morphologies effectively suppress interfacial charge recombination, thereby contributing to the improved photovoltaic performance of the devices.

**FIGURE 7:**
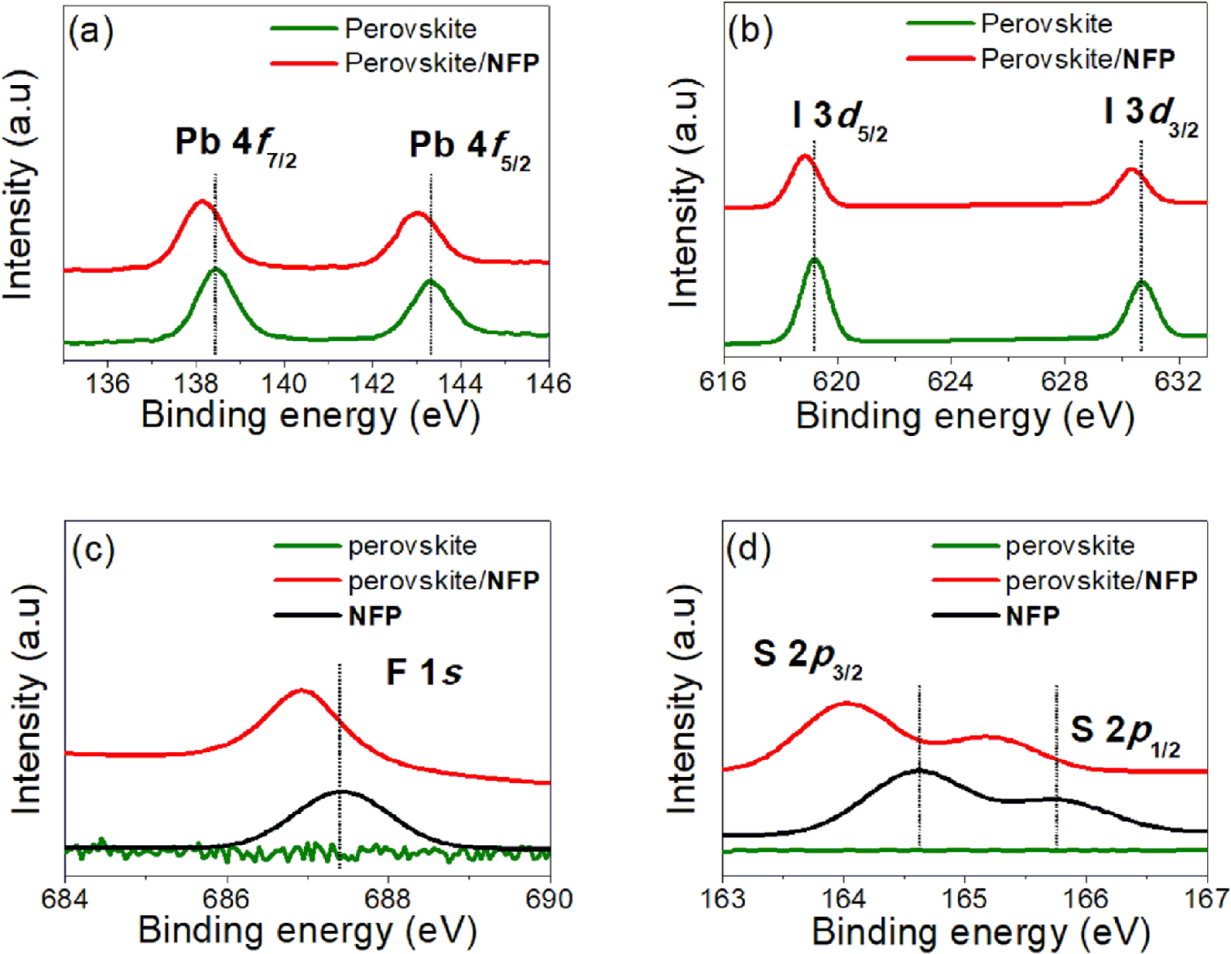
XPS signals of Pb 4f, I 3d, F 1s, and S 2p from a neat **NFP** film and an **NFP**‐coated perovskite film.

To gain further insight into the interfacial interactions between the HTMs and the perovskite layer, X‐ray photoelectron spectroscopy (XPS) measurements were performed on pristine perovskite films as well as perovskite films coated with **NPH**, **NPF**, and **NFP**, as shown in Figures 7, S52 and S53. The detailed experimental setup, fitting procedures, and uncertainty analysis are provided in the Supporting Information. For the pristine perovskite film, the Pb 4f and I 3d spectra exhibit characteristic doublets located at 143.3/138.4 eV and 630.7/619.2 eV, corresponding to the Pb 4f_5/2_/Pb 4f_7/2_ and I 3d_3/2_/I 3d_5/2_ core levels, respectively. After deposition of the HTM layers, both the Pb 4f and I 3d peaks shifted slightly toward lower binding energies, indicating that the HTMs modify the local electronic environment at the perovskite surface through interfacial electronic interactions. For the fluorinated HTMs, the F 1s spectra of the neat **NPF** and **NFP** films were compared with those of the corresponding HTM‐coated perovskite films. A slight shift toward lower binding energy was observed after contact with the perovskite surface (Figures 7c and S53c), suggesting that fluorine substitution also participates in the interfacial electronic redistribution. In addition, the S 2p spectra of neat **NPH**, **NPF**, and **NFP** films exhibited systematic shifts toward lower binding energies after deposition on the perovskite films. Taken together, these XPS results indicate the formation of an electronically coupled interface between the HTMs and the perovskite, which is consistent with enhanced interfacial coupling and reduced surface electronic defects. It should be noted that the lower‐binding‐energy shift of the S 2p signal should not be interpreted as direct evidence of a simple isolated S→Pb^2^^+^ coordination bond. The direction and magnitude of XPS binding‐energy shifts are influenced by several factors, including local charge redistribution, final‐state screening, molecular orientation, and the overall coordination environment at the interface. Therefore, the observed shifts are more appropriately interpreted as signatures of interfacial electronic redistribution rather than evidence of a single chemical bonding interaction. Accordingly, the simultaneous shifts of the Pb 4f, I 3d, S 2p, and F 1s signals collectively suggest that deposition of the HTMs modifies the electronic structure at the perovskite/HTM interface. For the fluorinated HTMs (**NPF** and **NFP**), fluorine substitution further contributes to this interfacial electronic coupling through its strong electron‐withdrawing character and local dipolar effects, thereby facilitating a more favorable interfacial electronic environment. Combined with the DFT calculations and the improved photovoltaic performance, these results support the conclusion that fluorination optimizes the interfacial electronic interactions between the HTMs and the perovskite, leading to more efficient charge extraction and suppressed interfacial non‐radiative recombination.

**FIGURE 8:**
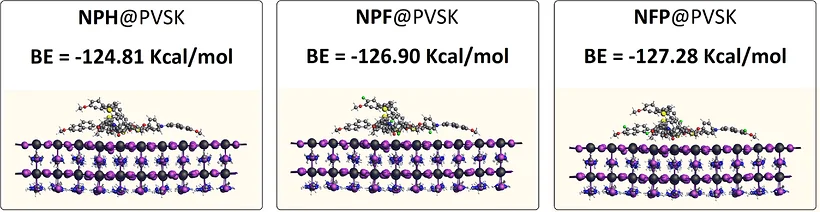
DFT‐simulated stacking configurations and binding energies (BEs) of the HTMs on the perovskite surface.

To elucidate the interfacial interactions between the various HTM molecules and the perovskite surface, DFT calculations were performed to evaluate the binding energies. As shown in Figure 8, the **NPH**, **NPF**, and **NFP** molecules adopt nearly planar orientations on the perovskite surface, in contrast to their free‐state conformations. This conformational adjustment is attributed to the coordination of sulfur atoms with Pb sites in the perovskite lattice. The calculated binding energies for **NPF** (−126.90 kcal mol^−^^1^) and **NFP** (−127.28 kcal mol^−^^1^) are slightly more negative than that of **NPH** (−124.81 kcal mol^−^^1^), which lacks fluorine substitution. Although the fluorinated derivatives exhibit slightly stronger adsorption than the non‐fluorinated analogue, the energy differences are relatively small (approximately 2–2.5 kcal mol^−^^1^). Therefore, these calculations should be regarded as providing qualitative evidence for a modest enhancement in interfacial affinity upon fluorination rather than definitive proof of substantially stronger interfacial binding or defect passivation. Furthermore, the calculations were performed on an idealized FAPbI_3_ surface and do not explicitly account for the chemically heterogeneous, mixed‐cation/mixed‐halide composition or defect‐containing interfaces employed in the experimental devices. Consequently, the DFT results are intended to complement, rather than quantitatively reproduce, the experimentally observed interfacial behavior. When considered together with the XPS analysis, charge‐transport measurements, and photovoltaic performance, the calculations support the conclusion that fluorination contributes to a more favorable interfacial electronic environment and facilitates efficient charge extraction.

**FIGURE 9:**
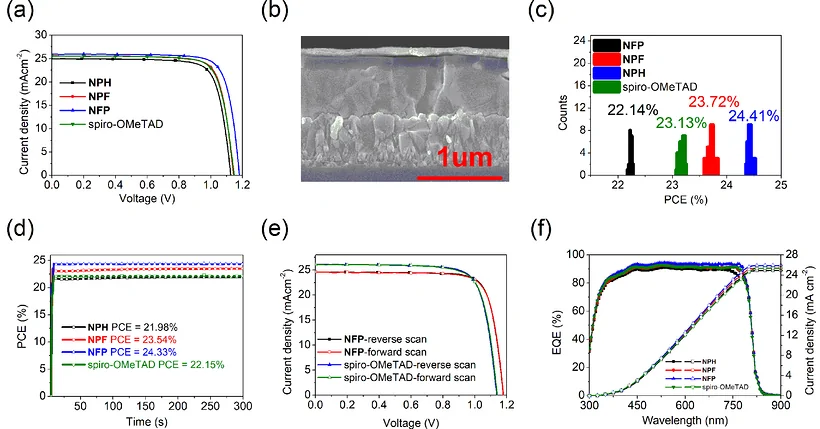
(a) *J–V* characteristics of the best‐performing PSCs with **NPH**, **NPF**, **NFP**, and spiro‐OMeTAD as HTMs. (b) Cross‐sectional SEM image of the PSC device with NFP as the HTM. (c) Statistical distribution of PCEs from 20 independent devices. (d) Stabilized power output for **NPH, NPF**, and **NFP** as well as spiro‐OMeTAD‐based PSCs. (e) *J–V* curves of large‐area (aperture area of 1.00 cm^2^) PSCs based on **NFP** and spiro‐OMeTAD. (f) EQE spectra and the integrated current of the PSCs with **NPH**, **NPF**, **NFP**, and spiro‐OMeTAD.

To evaluate the scalability of the developed HTMs, we implemented a fully blade‐coating‐based fabrication process for n–i–p PSCs. Unlike spin coating, blade coating enables precise control over film thickness and solvent evaporation dynamics, making it more suitable for large‐area and industrial‐scale fabrication. A key feature of this process is the incorporation of a vacuum‐assisted crystallization step, which rapidly removes residual solvents and induces instantaneous nucleation. This non‐equilibrium crystallization pathway results in highly uniform and compact perovskite films with reduced defect density. Furthermore, the blade‐coated HTM layer combined with vacuum flash treatment enhances interfacial contact and film homogeneity, contributing to improved device reproducibility and performance. This scalable processing strategy highlights the compatibility of the developed HTMs with practical manufacturing techniques. The schematic illustration of a fully blade‐coating–based fabrication process combined with vacuum‐assisted crystallization is provided in the Figure S54. The photovoltaic performance of PSCs employing **NPH**, **NPF**, and **NFP** was systematically evaluated. We fabricated a series of n–i–p‐structured devices with the configuration ITO/SnO_2_/Cs_0_._05_MA_0_._2_FA_0_._75_Pb(Br_0_._05_I_0_._95_)_3_/HTM/Ag. The current density–voltage (J–V) characteristics of the best‐performing devices are presented in Figure 9a, while the corresponding photovoltaic parameters are summarized in Table 2. For reference, spiro‐OMeTAD‐based devices were also fabricated under identical conditions. All HTMs were doped with 4‐tert‐butylpyridine (t‐BP) and lithium bis(trifluoromethanesulfonyl)imide (Li‐TFSI) to enhance charge transport and device stability. A representative cross‐sectional SEM image of a PSC incorporating **NFP** as the HTM is displayed in Figure 9b, showing thicknesses of approximately 135 nm for the **NFP** layer and 650 nm for the perovskite layer. The perovskite solar cells employing **NPH** as the HTM delivered a PCE of 22.28%, with a short‐circuit current density (*J*_SC_) of 24.98 mA cm^−^^2^, an open‐circuit voltage (*V*_OC_) of 1.13 V, and a fill factor (FF) of 78.93%. In comparison, the device incorporating the fluorinated **NPF** exhibited an enhanced PCE of 23.83%, accompanied by a *V*_OC_ of 1.17 V, a *J*_SC_ of 25.58 mA cm^−^^2^, and a FF of 79.62%. Remarkably, introducing fluorine atoms at the ortho position relative to the terminal methoxy group on the naphthalene ring of the terminal unit (**NFP**) further improved the photovoltaic characteristics. The **NFP**‐based device achieved a *V*_OC_ of 1.18 V, a *J*_SC_ of 25.94 mA cm^−^^2^, a FF of 80.02%, and an overall PCE of 24.49%, surpassing even that of the reference cell employing spiro‐OMeTAD (PCE: 23.19%, *V*_OC_: 1.15 V, *J*_SC_: 25.54 mA cm^−^^2^, FF: 78.97%). The progressive enhancements in *V*_OC_, *J*_SC_, FF, and PCE can be attributed to improved energy‐level alignment between the perovskite layer and the HTMs, increased hole mobility and extraction efficiency, smoother film morphology, and superior interfacial contact. Evidently, fluorine substitution on the naphthyl moiety of the HTM (**NFP**) plays a pivotal role in optimizing device performance. Importantly, negligible hysteresis was observed between forward and reverse J–V scans for devices based on **NPH**, **NPF**, **NFP**, and spiro‐OMeTAD (Figure S55) with hysteresis indices (HI) of 1.48%, 0.38%, 0.73%, and 0.04% for **NPH**, **NPF**, **NFP**, and spiro‐OMeTAD, respectively, calculated using the relation HI = (PCE_(__reverse__)_ – PCE_(__forward__)_)/PCE_(__reverse__)_. The statistical distribution of PCEs for 20 individual PSCs is presented in Figure 9c. The average PCEs achieved for devices employing **NPH**, **NPF**, **NFP**, and spiro‐OMeTAD as HTMs were 22.14%, 23.72%, 24.41%, and 23.13%, respectively. The narrow PCE distributions observed across these devices indicate excellent reproducibility. As shown in Figure 9d, the steady‐state power output was measured at the maximum power point (MPP) under one‐sun illumination for 300 s to evaluate the operational stability. The stabilized efficiencies of PSCs based on **NPH**, **NPF**, **NFP**, and spiro‐OMeTAD were determined to be 21.98%, 23.54%, 24.33%, and 22.15%, respectively, further confirming their reliable performance. To further demonstrate scalability, large‐area devices (1.00 cm^2^) were fabricated using blade coating. The **NFP**‐based devices maintain a high PCE of 23.28% (*V*_OC_: 1.17 V, *J*_SC_: 25.33 mA cm^−^^2^, FF: 78.54%), significantly outperforming the spiro‐OMeTAD counterpart (20.22%). Additionally, we examined dopant‐free PSCs utilizing **NPH**, **NPF**, **NFP**, and spiro‐OMeTAD as HTMs. These devices yielded PCEs of 11.64%, 13.59%, 15.03%, and 13.15%, respectively, under reverse scan conditions (Figure S56 and Table S2). These results suggest that the incorporation of dopants remains essential for enhancing the photovoltaic performance of this class of HTMs. Moreover, as illustrated in Figure 9f, the devices employing **NPH**, **NPF**, **NFP**, and spiro‐OMeTAD exhibit nearly identical integrated *J_SC_* values. Because the electrical conductivity of doped arylamine‐based HTMs strongly depends on the formation of oxidized species, the influence of oxidation time on device performance was further investigated. As shown in Figure S57, the PCEs of PSCs employing **NPH**, **NPF**, **NFP**, and spiro‐OMeTAD gradually increased with oxidation time and reached their maximum values after approximately 2 h of exposure to ambient atmosphere. Further oxidation did not provide additional performance improvement, indicating that an appropriate oxidation level is required to achieve optimal hole transport. Notably, the three newly developed HTMs exhibited oxidation behaviors comparable to that of spiro‐OMeTAD, suggesting that the introduction of asymmetric fluorinated triphenylamine units does not significantly alter the oxidation process required for efficient device operation.

**TABLE 2:** Photovoltaic parameters extracted from *J–V* measurements of PSCs based on various HTMs.

HTM	Scan direction		*V*_oc_ [V]	*J*_sc_ [mA cm^−2^]	FF (%)	PCE_max_ [%]
**NPH**	Forward		1.12	24.94	78.59	21.95
	Reverse	Best	1.13	24.98	78.93	22.28
		Average	1.12 ± 0.005	24.95 ± 0.03	78.90±0.03	22.14 ± 0.07
**NPF**	Forward		1.17	25.56	79.38	23.74
	Reverse	Best	1.17	25.58	79.62	23.83
		Average	1.15 ± 0.009	25.56 ± 0.02	79.60±0.02	23.72 ± 0.05
**NFP**	Forward		1.18	25.89	79.59	24.31
	Reverse	Best	1.18	25.94	80.02	24.49
		Average	1.17 ± 0.003	25.97 ± 0.01	80.01±0.01	24.41 ± 0.02
	Forward		1.15	25.52	78.99	23.18
Spiro‐OMeTAD	Reverse	Best	1.15	25.54	78.97	23.19
		Average	1.14 ± 0.008	25.52 ± 0.02	78.95 ± 0.02	23.13 ± 0.05

**FIGURE 10:**
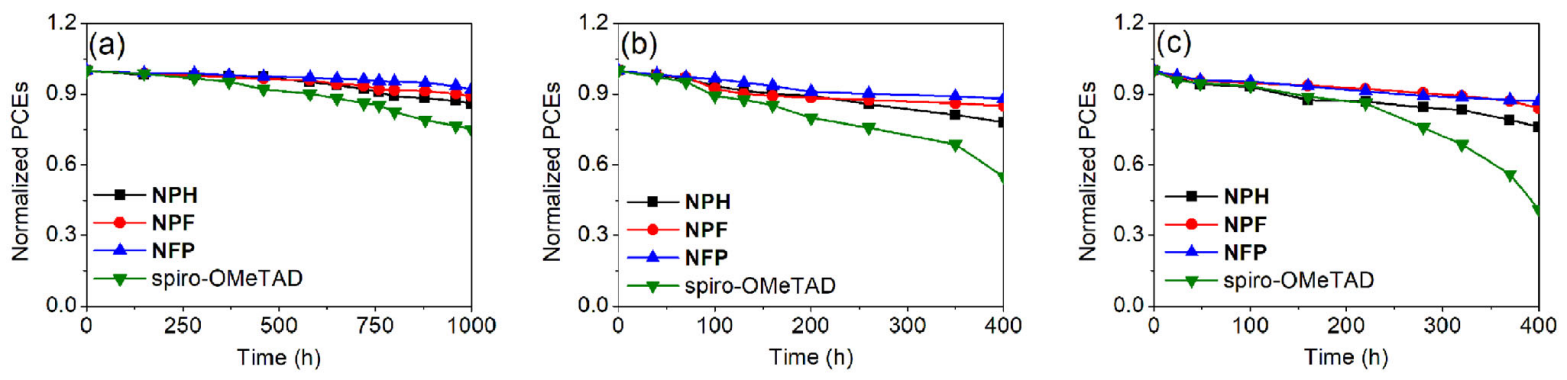
Normalized long‐term device stability of **NPH**‐, **NPF**‐, **NFP**‐, and spiro‐OMeTAD‐based PSCs: (a) under ambient conditions (20–25°C); (b) under heating conditions (85°C); and (c) under continuous one‐sun illumination at 40°C in N_2_.

The HTM in n–i–p‐structured PSCs critically influences device stability. Unencapsulated PSCs employing **NPH**, **NPF**, **NFP**, and spiro‐OMeTAD as HTMs were evaluated under 40% relative humidity. As shown in Figure 10a, devices with **NPH**, **NPF**, and **NFP** retained 86%, 89%, and 92% of their initial PCE, respectively, after 1000 h of dark storage, whereas the spiro‐OMeTAD‐based device retained only 75%. The superior stability of the fluorinated HTMs (**NPF** and **NFP**) is attributed to enhanced hydrophobicity of the overlying layers. Water contact angle measurements (Figure S58) revealed values of 91°, 96°, 99°, and 73° for **NPH**‐, **NPF**‐, **NFP**‐, and spiro‐OMeTAD‐coated films, respectively, confirming that fluorination effectively improves moisture resistance and contributes to long‐term stability. In addition, **NPF**‐ and **NFP**‐based devices exhibited superior thermal endurance at 85°C, maintaining 85% and 88% of their initial PCEs after 400 h, compared to 78% for **NPH** and 55% for spiro‐OMeTAD (Figure 10b). Under continuous illumination (Figure 10c), devices incorporating **NPF** and **NFP** retained over 80% of their initial efficiency after 400 h, outperforming the **NPH** (76%) and spiro‐OMeTAD (41%) counterparts. The improved photostability of the fluorinated HTMs is ascribed to their more stable amorphous morphology, consistent with DSC analysis (Figure S47). Collectively, these findings demonstrate that fluorine substitution in HTMs markedly enhances the environmental stability, thermal stability, and photostability of PSCs, leading to durable, high‐performance devices. Furthermore, a detailed comparison of the previously reported **spiro‐1** and **spiro‐2** with **NPH**, **NPF**, and **NFP**, including their molecular structures, synthetic routes, yields, device‐processing methods, photovoltaic performance, and stability, is provided in Table S3.

## 3 Conclusions

In summary, we have developed a series of asymmetrically substituted spiro‐type HTMs featuring phenylnaphthyl terminal groups with controlled fluorination through a facile and efficient synthetic strategy, enabling rapid construction of the spiro framework under mild conditions. This synthetic simplicity provides a practical advantage over conventional multistep HTM systems and enhances the potential for scalable material production. Through the deliberate integration of molecular asymmetry and fluorination, we achieve simultaneous modulation of intermolecular packing, energy‐level alignment, and interfacial interactions. Compared to the nonfluorinated analogue **NPH**, the fluorinated derivatives **NPF** and **NFP** exhibit deeper HOMO energy levels, improved hole mobility, and enhanced π–π stacking, resulting in more efficient hole extraction and suppressed non‐radiative recombination. These improvements are supported by PL, TRPL, and SCLC measurements, as well as DFT calculations, which reveal strengthened interfacial binding and more effective defect passivation at the perovskite/HTM interface. In addition, GIWAXS analyses confirm that fluorination promotes tighter molecular packing, likely driven by enhanced dipole–dipole interactions, thereby contributing to improved charge transport properties. Importantly, beyond molecular design, we further demonstrate a scalable blade‐coating fabrication strategy combined with vacuum‐assisted crystallization, enabling precise control over film formation and crystallization kinetics. This processing approach produces uniform and compact films with reduced defect density and improved interfacial contact, which are essential for achieving high‐performance and large‐area devices. As a result, PSCs incorporating the optimized HTM (**NFP**) achieve a high PCE of 24.49%, surpassing that of the benchmark spiro‐OMeTAD, while maintaining an impressive PCE of 23.28% in large‐area (1.00 cm^2^) devices. In addition, **NFP**‐based devices exhibit enhanced operational stability under ambient, thermal, and continuous illumination conditions, which can be attributed to improved interfacial passivation, molecular packing, and hydrophobicity induced by fluorination. These results establish fluorination and its substitution position as effective design parameters for tuning the properties of asymmetric spiro‐CPDT HTMs. This integrated approach provides a generalizable framework for bridging the gap between high‐performance materials and practical device manufacturing, offering a viable pathway toward the commercialization of perovskite solar cells.

## Author Contributions

**Kun‐Mu Lee**: supervision, formal analysis, project administration. **Hsiao‐Chi Hsieh**: investigation, data curation, formal analysis. **Yu‐Che Lin**: investigation, formal analysis, data curation, methodology. **Cheng‐Ting Tsai**: methodology, data curation, formal analysis. **Pei‐Chen Chu**: methodology, formal analysis, data curation. **Yu‐Hsun Chen**: methodology, formal analysis, data curation. **Kang‐Ling Liau**: software, data curation. **Ya‐Ho Chang**: investigation, formal analysis, data curation. **Xiu‐Ping Lin**: methodology, formal analysis, data curation. **Sie‐Rong Li**: data curation, formal analysis. **Wei‐Hao Chiu**: investigation, data curation, formal analysis. **Chih‐Min Wang**: supervision, formal analysis. **Yan‐Duo Lin**: writing – original draft, writing – review and editing, project administration, supervision.

## Conflicts of Interest

The authors declare no conflicts of interest.

## Supporting information




**Supporting File 1**: advs77623‐sup‐0001‐SuppMat.docx.

**Supporting File 2**: advs77623‐sup‐0002‐MovieS1.mp4.

## Data Availability

The data that support the findings of this study are available in the Supporting Information for this article.
