# Supplementary material for: Asymmetric Fluorinated Spiro Hole‐Transport Materials Enabled by a Rapid Core‐Forming Strategy for Blade‐Coated Perovskite Solar Cells
Source: Adv Sci (Weinh). 2026 Sep 8:e77623. Online ahead of print. doi: 10.1002/advs.77623 (PMC13554398; doi:10.1002/advs.77623)
Supplement: Supplementary file 1 [file ADVS-9999-e77623-s001.docx]

Supporting Information

Asymmetric Fluorinated Spiro Hole-Transport Materials Enabled by a Rapid Core-Forming Strategy for Blade-Coated Perovskite Solar Cells

Kun-Mu Lee,*^a,b,c,d^ Xiu-Ping Lin,^e^ Ya-Ho Chang,*^f^ Cheng-Ting Tsai,^e^ Pei-Chen Chu,^e^ Yu-Hsun Chen,^e^ Wei-Hao Chiu,^b^ Yu-Che Lin,^e^ Sie-Rong Li,^g^ Hsiao-Chi Hsieh,*^h^ Kang-Ling Liau,^i^ Shih-I Lu,*^e^ Chih-Min Wang*^f^ and Yan-Duo Lin*^e^

^a^ Department of Chemical and Materials Engineering, Chang Gung University, Taoyuan 33302, Taiwan

^b^ Center for Sustainability and Energy Technologies, Chang Gung University, Taoyuan 33302, Taiwan

^c^ Division of Neonatology, Department of Pediatrics, Chang Gung Memorial Hospital, Linkou, Taoyuan 33305, Taiwan

^d^ College of Environment and Resources, Ming Chi University of Technology, New Taipei City 24301, Taiwan

^e^ Department of Chemistry, Soochow University, Taipei 11102, Taiwan

^f^ Department of Bioscience and Biotechnology, National Taiwan Ocean University, 202, Keelung, 20224, Taiwan

^g^ Institute of Chemistry, Academia Sinica, Taipei 115024, Taiwan

^h^ Department of Chemical and Materials Engineering, Tamkang University, New Taipei City 24301, Taiwan

^i^ Department of Chemistry, National Central University, Taoyuan 32001, Taiwan

† Supporting Information (SI) is available. For other electronic formats, see DOI: XXX

Email: ydlin@scu.edu.tw

**Materials and Reagents**

All solvents and chemicals were purchased from Aldrich, with the purity more than 98%. The thin-layer chromatography (TLC) was conducted with Merck KGaA precoated TLC Silica gel 60F254 on aluminum sheets. Flash column chromatography was performed on glass columns packed with silica gel (Silicycle UltraPure SilicaFlash P60, 40−63 mm (230−400 mesh)). Unless otherwise specified, all reactions and manipulations were carried out under nitrogen atmosphere. Solvents of reagent grade were used for syntheses and those of spectroscopy grade for spectra measurements. Solvents were dried by standard procedures.

**Characterization**

The ^1^H and ^13^C NMR spectra were recorded on a Bruker 400 or 500 MHz spectrometer. Fast atom bombardment (FAB) mass spectra were recorded on a Jeol JMS 700 double-focusing spectrometer. The UV spectra were measured on a Jasco V-530 double beam spectrophotometer. Fluorescence spectra were recorded on a Hitachi F-4500 fluorescence spectrophotometer. Cyclic voltammetry experiments were performed with a CHI-621A electrochemical analyzer. All measurements were carried out at room temperature with a conventional three-electrode configuration that consisted of a platinum working electrode, an auxiliary electrode, and a non-aqueous Ag/AgNO_3_ reference electrode. The SEM images were obtained by using a field-emission scanning electron microscope (JEOL-7401). A Nano-Scope NS3A system (Digital Instrument) was used to obtain the AFM images of the surface morphologies and thicknesses of various thin films. X-ray photoelectron spectroscopy (XPS) was collected with a VG ESCA Scientific Theta Probe system with a monochromatic Al Kα source (1486.6 eV) operated under ultra-high vacuum (< 2.5 × 10^−10^ Torr) and analyzed by XPSPEAKS41 software. In addition, charge neutralization was used during all measurements. All binding energies were calibrated by shifting the C 1s peak to 284.8 eV. The PL excitation wavelength used in this work was 532 nm, and the detection center wavelength was set at ~780 nm.

**Solar cell fabrication.**

**Device fabrication of the perovskite solar cells (PSCs)**

Patterned indium tin oxide (ITO) glass substrates (5 × 5 cm²) were sequentially cleaned by ultrasonication in detergent solution, deionized water, acetone, and isopropanol for 30 min each, followed by drying under a nitrogen stream. The SnO₂ electron-transporting layer (ETL) was deposited onto the ITO substrates via blade coating using a diluted SnO₂ dispersion. The substrate temperature was maintained at 40 °C during coating, with a blade gap of 30 μm and a coating speed of 10 mm s⁻¹. The resulting films were annealed at 150 °C for 30 min to ensure complete film formation. Subsequently, the SnO₂-coated substrates were treated with UV–ozone for 15 min to improve surface wettability and remove residual organic contaminants. The precursor solution (1.25 M) was prepared by dissolving 576.3 mg PbI₂, 161.2 mg FAI, 14 mg MABr, 16.2 mg CsI, and 19.8 mg MAI in 0.8 mL DMF and 0.2 mL DMSO, followed by stirring at 60 °C for 8 h. The perovskite layer was deposited via blade coating using a blade gap of 50 μm, a coating speed of 20 mm s⁻¹, a dispensing volume of 40 μL, and a substrate temperature of 28 °C. Immediately after coating, a vacuum flash treatment was applied in a sealed chamber (5 Pa) for 45 s, inducing rapid solvent extraction and crystallization, as indicated by the color transition of the film to dark brown. The films were subsequently annealed at 150 °C for 10 min. The hole-transporting materials (HTMs) was deposited on top of the perovskite film by blade coating with a blade gap of 30 μm and a coating speed of 10 mm s⁻¹, followed by a second vacuum flash treatment (5 Pa, 25 s) to improve film uniformity and interfacial contact. Finally, a 100 nm thick Ag electrode was thermally evaporated under high vacuum (< 1 × 10⁻⁴ Pa) to complete the device structure.

**IPCE evaluation of the perovskite solar cells (PSCs)**

An IPCE spectrometer (ENLI Technology Co. Ltd., QE-R) calibrated with a single-crystal silicon reference cell was used for the incident monochromatic photon-to-current conversion efficiency (IPCE) measurements. An AM 1.5G solar simulator (ENLI Technology Co. Ltd., SS-F7-3A) was used as the irradiation light source for the characteristic current density-voltage (J-V) measurements. The intensity of the simulated sunlight was calibrated to 100 mW/cm^2^. The J-V characteristics of the cell under an illumination of AM 1.5G simulated sunlight were obtained by applying the external potential bias to the cell and measuring the photocurrent output with a precision power measurement equipment (KEYSIGHT Technologies, B2901A).

**Stability Testing Environment**

The long-term stability of the unencapsulated perovskite solar cells (PSCs) employing **NPH**, **NPF**, **NFP**, and spiro-OMeTAD as hole-transporting materials (HTMs) was evaluated under controlled conditions. The devices were stored and tested at room temperature (20–25 °C) with a relative humidity of under 40%.

**Mobility measurements**

In order to assess the potential of **NPH**, **NPF**, **NFP**, and spiro-OMeTAD as hole-transport materials, hole-only devices were fabricated with the device structure of ITO/PEDOT:PSS/HTM/Al. Hole mobilities were calculated by the space-charge-limited current (SCLC) method using the Mott–Gurney law, by fitting experimental data to equation (1) in the voltage range where the obtained slope in the double log plot is equal to 2.

(1)

In equation (1), *J* is the current density, ε_0_ is the permittivity of free space (8.85 x10^-12^ F m^-1^), ε_r_ is the relative permittivity of the material (approaching 3 for organic semiconductors), μ_h_ is the hole mobility, *V* is the applied voltage and *d* is the thickness of the active layer.

The hole-only devices were fabricated by spin-coating PEDOT:PSS (Clevios P, VP Al4083) onto pre-cleaned, patterned indium tin oxide (ITO) substrates (15 Ω/square) (Kintec). A film of the HTM was spin-coated on top from chloroform solution with a concentration of 40 mg mL^-1^. The film thickness was varied by using different spin-coating speeds. As a counter electrode, Al was deposited on top by vacuum evaporation. The current density–voltage curves of the devices were recorded with a Keithley 2400 Source meter.

**GIWAXS** **measurements**

Grazing-incidence wide-angle X-ray scattering (GIWAXS) measurements were performed at beamlines 01C, 17A, and 13A of the Taiwan Light Source at the National Synchrotron Radiation Research Center (NSRRC). The sample-to-detector distance was approximately 240 mm, and the incident angle was fixed at 0.2°. The out-of-plane π–π stacking diffraction peaks were fitted with Gaussian functions to determine their full width at half maximum (FWHM, Δq). The crystalline coherence length (Lc) was estimated according to Lc = 0.9×2π/Δq, where Δq is the full width at half maximum (FWHM) of the diffraction peak in reciprocal space, and 0.9 is the Scherrer shape factor. Near-edge X-ray absorption fine structure (NEXAFS) spectroscopy was performed at beamline BL24A of the Taiwan Light Source at NSRRC using the total electron yield (TEY) detection mode.

**XPS Measurement and Peak Fitting Procedures:**

X-ray photoelectron spectroscopy (XPS) measurements were collected using a VG ESCA Scientific Theta Probe system with a monochromatic Al Kα source (1486.6 eV) operated under ultra-high vacuum (< 2.5 × 10^-10^ Torr) with charge neutralization. All binding energies were calibrated by referencing the adventitious C 1s peak to 284.8 eV. Spectral deconvolution and peak fitting were analyzed using XPSPEAK41 software with a Shirley-type background subtraction and Gaussian–Lorentzian line shapes. The overall measurement and fitting uncertainty for peak positions is estimated to be ± 0.05 eV.

**Computation method**

Ground-state geometries and the corresponding vertical excitation energies of the **NPH**, **NPF**, and **NFP** molecules in the THF were studied computationally by performing CAM-B3LYP [2] and TD-CAM-B3LYP calculations, respectively, with the 6-31G(d,p) basis set via the Gaussian 16 software package [3]. The integral equation formalism polarizable continuum model (IEFPCM) was employed to include the solvent effect [4].

To further study the interaction between the perovskite (FAPbI_3_, here) and the molecules of study, we conducted geometry optimization within the context of periodic boundary conditions (PBC) in which one molecule of **NPH**, **NPF**, and **NFP** was placed over the FAPbI_3_ surface. The M3GNet machine learning potential [5] with the PBE-D3(BJ) dispersion [6] and the density functional-based tight binding method using the GFN1-xTB [7] parametrization of the extended tight-binding (xTB) model Hamiltonian were employed to perform geometry optimizations and binding energy calculations, respectively. We used the Amsterdam Modeling Suite (AMS) [8] to perform the calculations. A slab model is constructed with periodic boundary conditions applied in-plane, while a sufficiently large vacuum layer is introduced perpendicular to the slab to prevent artificial interactions between periodic images. Energy convergence tests confirm that this vacuum thickness is sufficient to prevent artificial coupling. This setup allows for accurate surface calculations while maintaining computational efficiency. To mimic the FAPbI_3_ surface slabs, we employed the (001) surface of 48.6 Å × 48.6 Å with a vacuum layer of 100 Å.

**Scheme S1.** Synthetic procedures for **NPH**, **NPF**, and **NFP.**

*Synthesis of* ***2***: A heterogeneous mixture of THF (10 mL), **1** (0.5 g, 2.6 mmol), and N-Bromosuccinimide (1.16 g, 2.5 eq) under room temperature was stirred for 1 h. Evaporation of the solvent gave a crude product, which was purified by silica gel column chromatography using CH_2_Cl_2_/hexane (1/5) as eluent to afford the desired product as a purple solid in 62% yield. mp 83-84 °C

*Synthesis of* ***3***: A heterogeneous mixture of CH_2_Cl_2_ (10 mL), **2** (0.5 g, 2.6 mmol), and triisopropylphosphite (0.65 g, 2 eq) under room temperature was stirred for 1 h. Evaporation of the solvent gave a crude product, which was purified by recrystallization to afford the desired product as a yellow solid in 50% yield. mp 96-97 °C

*Synthesis of* ***10****:* A heterogeneous mixture of anhydrous Toluene (6 mL), **4** (0.5 g, 2.1 mmol), **5** (0.2 g, 1.7 mmol), Pd_2_(dba)_3_ (0.02 g, 1 mol %), Pd(t-bu)_3_HBF_4_ (0.02 g, 3 mol %) and NaOtBu (0.2 g, 1.2eq), under argon was stirred under reflux for 12 h. Evaporation of the solvent gave a crude product, which was purified by silica gel column chromatography using ethyl acetate/hexane (1/30) as eluent to afford the desired product as a white solid in 82% yield. mp 129-130 °C. ^1^H NMR (500 MHz, CDCl_3_): δ 7.60 (d, *J* = 8.5 Hz, 1H), 7.50 (d, *J* = 8.5 Hz, 1H), 7.19 (s, 1H), 7.11–7.09 (m, 3H), 7.07–7.05 (m, 2H), 6.87 (d, *J* = 9.0 Hz, 2H), 5.54 (br s, 1H, NH), 3.87 (s, 3H, OMe), 3.80 (s, 3H, OMe) ppm; ^13^C NMR (125 MHz, CDCl_3_): δ 156.03, 155.34, 141.19, 136.51, 130.27, 129.70, 128.10, 128.01, 121.89, 120.00, 119.19, 114.96, 110.51, 106.27, 55.83, 55.53 ppm. HRMS (FAB) m/z: [M]^+^ calcd for C_18_H_17_NO_2_, 279.1259; found, 279.1261.

*Synthesis of* ***11***: A heterogeneous mixture of anhydrous Toluene (6 mL), **6** (0.5 g, 2.1 mmol), **7** (0.3 g, 2.3 mmol), Pd_2_(dba)_3_ (0.03 g, 2 mol %), Pd(t-bu)_3_HBF_4_ (0.02 g, 3 mol %) and NaOtBu (0.4 g, 2eq), under argon was stirred under reflux for 12 h. Evaporation of the solvent gave a crude product, which was purified by silica gel column chromatography using ethyl acetate/hexane (1/20) as eluent to afford the desired product as a white solid in 62% yield. mp 101-102 °C. ^1^H NMR (500 MHz, CDCl_3_): δ 7.63 (d, *J* = 9.0 Hz, 1H), 7.54 (d, *J* = 9.0 Hz, 1H), 7.28 (d, *J* = 2.0 Hz, 1H), 7.13 (dd, *J* = 9.0, 2.5 Hz, 1H), 7.09 (dd, *J* = 9.0, 2.5 Hz, 1H), 7.06 (d, *J* = 2.5 Hz, 1H), 6.92–6.87 (m, 2H), 6.81–6.78 (m, 1H), 5.58 (br s, 1H, NH), 3.88 (s, 3H, OMe), 3.86 (s, 3H, OMe) ppm; ^13^C NMR (125 MHz, CDCl_3_): δ 156.42, 154.19, 152.24, 142.62, 142.53, 139.65, 137.84, 137.77, 130.28, 130.09, 128.24, 128.20, 120.61, 119.37, 115.21, 115.19, 114.44, 114.42, 112.41, 107.87, 107.70, 106.23, 57.25, 55.54 ppm; ^19^F NMR (470 MHz, CDCl_3_): δ –133.05 (m, 1F) ppm. HRMS (FAB) m/z: [M]^+^ calcd for C_18_H_16_FNO_2_, 297.1165; found, 297.1170.

*Synthesis of* ***12****:* A heterogeneous mixture of anhydrous Toluene (6 mL), **8** (0.5 g, 1.96 mmol), **9** (0.3 g, 2.2 mmol), Pd_2_(dba)_3_ (0.04 g, 2 mol %), Pd(t-bu)_3_HBF_4_ (0.02 g, 3 mol %) and NaOtBu (0.38 g, 2eq), under argon was stirred under reflux for 12 h. Evaporation of the solvent gave a crude product, which was purified by silica gel column chromatography using ethyl acetate/hexane (1/30) as eluent to afford the desired product as a yellow solid in 86% yield. mp 91-92 °C. ^1^H NMR (500 MHz, CDCl_3_): δ 7.89 (d, *J* = 8.5 Hz, 1H), 7.30 (d, *J* = 9.0 Hz, 1H), 7.19 (d, *J* = 8.5 Hz, 1H), 7.16–7.10 (m, 4H), 6.90 (d, *J* = 9.0 Hz, 2H), 5.61 (br s, 1H, NH), 3.96 (s, 3H, OMe), 3.81 (s, 3H, OMe) ppm; ^13^C NMR (125 MHz, CDCl_3_): δ 155.76, 148.59, 146.62, 142.35, 140.93, 140.86, 135.57, 131.23, 131.20, 122.76, 122.05, 122.01, 121.17, 121.13, 120.08, 119.62, 119.51, 117.19, 114.95, 108.38, 108.37, 58.23, 55.76 ppm; ^19^F NMR (470 MHz, CDCl_3_): δ –146.96 (d, *J* = 9.4 Hz, 1F) ppm; HRMS (FAB) m/z [M]^+^ calcd for C_18_H_16_FNO_2_: 297.1165; found: 297.1170.

*Synthesis of* ***13****:* A heterogeneous mixture of anhydrous Toluene (6 mL), **10** (0.5 g, 1.68 mmol), 1-Bromo-4-iodobenzene (0.7 g, 2.5 mmol), Pd_2_(dba)_3_ (0.05 g, 3 mol %), DPPF (0.07 g, 6 mol %) and NaOtBu (0.3 g, 2eq), under argon was stirred under reflux for 12 h. Evaporation of the solvent gave a crude product, which was purified by silica gel column chromatography using ethyl acetate/hexane (1/80) as eluent to afford the desired product as a yellow solid in 67% yield. mp 122-123 °C. ^1^H NMR (500 MHz, CDCl_3_): δ 7.62 (d, *J* = 9.0 Hz, 1H), 7.50 (d, *J* = 10.0 Hz, 1H), 7.35 (s, 1H), 7.29–7.26 (m, 2H), 7.22–7.21 (m, 1H), 7.09–7.06 (m, 4H), 6.91–6.89 (m, 2H), 6.85–6.83 (m, 2H), 3.89 (s, 3H, OMe), 3.80 (s, 3H, OMe) ppm; ^13^C NMR (125 MHz, CDCl_3_): δ 157.15, 156.51, 147.74, 143.53, 140.59, 132.14, 131.27, 129.92, 128.63, 128.02, 127.27, 124.94, 123.64, 120.75, 119.24, 115.07, 113.66, 106.07, 55.67, 55.53 ppm. HRMS (FAB) m/z: [M]^+^ calcd for C_24_H_20_BrNO_2_, 433.0677; found, 433.0686.

*Synthesis of* ***14****:* A heterogeneous mixture of anhydrous Toluene (6 mL), **11** (0.5 g, 1.68 mmol), 1-Bromo-4-iodobenzene (0.7 g, 2.5 mmol), Pd_2_(dba)_3_ (0.05 g, 3 mol %), DPPF (0.07 g, 6 mol %) and NaOtBu (0.3 g, 2eq), under argon was stirred under reflux for 12 h. Evaporation of the solvent gave a crude product, which was purified by silica gel column chromatography using ethyl acetate/hexane (1/80) as eluent to afford the desired product as a yellow solid in 69% yield. mp 110-111 °C. ^1^H NMR (500 MHz, CDCl_3_): δ 7.63 (d, *J* = 8.5 Hz, 1H), 7.51 (d, J = 8.5 Hz, 1H), 7.35 (s, 1H), 7.30 (d, J = 9.0 Hz, 2H), 7.19 (dd, J = 8.5, 2.0 Hz, 1H), 7.09 (d, J = 10.5 Hz, 2H), 6.92–6.80 (m, 5H), 3.89 (s, 3H, OMe), 3.87 (s, 3H, OMe) ppm; ^13^C NMR (125 MHz, CDCl_3_): δ 157.38, 153.91, 151.95, 147.22, 144.31, 144.22, 143.00, 141.15, 141.09, 132.35, 131.61, 129.88, 128.72, 128.25, 125.08, 124.34, 121.49, 120.68, 120.66, 119.40, 114.57, 114.44, 113.59, 113.43, 106.07, 56.83, 55.55 ppm; ^19^F NMR (470 MHz, CDCl_3_): δ –132.57 (m, 1F) ppm. HRMS (FAB) m/z: [M]^+^ calcd for C_24_H_19_BrFNO_2_, 451.0583; found, 451.0578.

*Synthesis of* ***15****:* A heterogeneous mixture of anhydrous Toluene (6 mL), **12** (0.5 g, 1.68 mmol), 1-Bromo-4-iodobenzene (0.7 g, 2.5 mmol), Pd_2_(dba)_3_ (0.05 g, 3 mol %), DPPF (0.07 g, 6 mol %) and NaOtBu (0.3 g, 2eq), under argon was stirred under reflux for 12 h. Evaporation of the solvent gave a crude product, which was purified by silica gel column chromatography using ethyl acetate/hexane (1/70) as eluent to afford the desired product as a yellow solid in 81% yield. mp 53-54 °C. ^1^H NMR (500 MHz, CDCl_3_): δ 7.87 (d, *J* = 9.0 Hz, 1H), 7.30–7.26 (m, 3H), 7.24–7.22 (m, 2H), 7.19 (t, *J* = 8.5 Hz, 1H), 7.05 (d, *J* = 9.0 Hz, 2H), 6.90 (d, *J* = 9.0 Hz, 2H), 6.84 (d, *J* = 9.0 Hz, 2H), 3.97 (s, 3H, OMe), 3.79 (s, 3H, OMe) ppm; ^13^C NMR (125 MHz, CDCl_3_): δ 156.79, 148.32, 147.36, 146.35, 144.61, 142.20, 142.12, 140.27, 132.31, 130.70, 130.67, 127.53, 124.76, 124.31, 122.82, 122.79, 121.03, 120.98, 119.00, 116.94, 115.18, 114.43, 58.08, 55.70 ppm; ^19^F NMR (470 MHz, CDCl_3_): δ –146.78 (d, *J* = 9.4 Hz, 1F) ppm; HRMS (FAB) m/z [M]^+^ calcd for C_24_H_19_BrFNO_2_: 451.0583; found: 451.0590.

*Synthesis of* ***16****:* A heterogeneous mixture of anhydrous Toluene (12 mL), **13** (0.5 g, 1.15 mmol), Bis(pinacolato)diborn (0.35 g, 1.38 mmol), Pd(PPh_3_)_2_Cl_2_ (0.02 g, 6 mol %) and KOAc (0.01 g, 3 eq), under argon was stirred under reflux for 12 h. Evaporation of the solvent gave a crude product, which was purified by silica gel column chromatography using ethyl acetate/hexane (1/30) as eluent to afford the desired product as a yellow solid in 60% yield. mp 118-119 °C. ^1^H NMR (500 MHz, CDCl_3_): δ 7.63–7.59 (m, 3H), 7.48 (d, *J* = 9.5 Hz, 1H), 7.38 (s, 1H), 7.24–7.22 (m, 1H), 7.09–7.05 (m, 4H), 6.96 (d, *J* = 8.5 Hz, 2H), 6.83 (d, *J* = 9.0 Hz, 2H), 3.88 (s, 3H, OMe), 3.79 (s, 3H, OMe), 1.31 (s, 12H, Bpin) ppm; ^13^C NMR (125 MHz, CDCl_3_): δ 157.21, 156.60, 151.26, 143.46, 140.57, 136.02, 131.45, 129.94, 128.74, 127.94, 127.72, 125.55, 121.60, 120.34, 119.18, 115.01, 106.08, 83.70, 55.69, 55.57, 25.06 ppm. HRMS (FAB) m/z: [M]^+^ calcd for C_30_H_32_BNO_4_, 481.2424; found, 481.2420.

*Synthesis of* ***17****:* A heterogeneous mixture of anhydrous Toluene (12 mL), **14** (0.5 g, 1.11 mmol), Bis(pinacolato)diborn (0.34 g, 1.33 mmol), Pd(PPh_3_)_2_Cl_2_ (0.02 g, 6 mol %) and KOAc (0.01 g, 3 eq), under argon was stirred under reflux for 12 h. Evaporation of the solvent gave a crude product, which was purified by silica gel column chromatography using ethyl acetate/hexane (1/30) as eluent to afford the desired product as a yellow solid in 80% yield. mp 134-135 °C. ^1^H NMR (500 MHz, CDCl_3_): δ 7.67–7.61 (m, 3H), 7.50 (d, *J* = 10.0 Hz, 1H), 7.39 (s, 1H), 7.23–7.20 (m, 1H), 7.10–7.07 (m, 2H), 6.99 (d, *J* = 8.0 Hz, 2H), 6.91 (s, 1H), 6.89–6.84 (m, 2H), 3.89 (s, 3H, OMe), 3.87 (s, 3H, OMe), 1.32 (s, 12H, Bpin) ppm; ^13^C NMR (125 MHz, CDCl_3_): δ 157.39, 153.88, 151.92, 150.73, 144.42, 144.33, 142.98, 141.10, 141.03, 136.11, 131.71, 129.89, 128.79, 128.14, 125.57, 122.12, 121.24, 121.22, 121.06, 119.31, 114.33, 114.31, 114.10, 113.94, 106.07, 83.77, 56.80, 55.56, 25.06 ppm; ^19^F NMR (470 MHz, CDCl_3_): δ –132.85 (m, 1F) ppm. HRMS (FAB) m/z: [M]^+^ calcd for C_30_H_31_BFNO_4_, 499.2330; found, 499.2322.

*Synthesis of* ***18****:* A heterogeneous mixture of anhydrous Toluene (12 mL), **15** (0.5 g, 1.11 mmol), Bis(pinacolato)diborn (0.34 g, 1.33 mmol), Pd(PPh_3_)_2_Cl_2_ (0.02 g, 6 mol %) and KOAc (0.01 g, 3 eq), under argon was stirred under reflux for 12 h. Evaporation of the solvent gave a crude product, which was purified by silica gel column chromatography using ethyl acetate/hexane (1/20) as eluent to afford the desired product as a yellow solid in 60% yield. mp 90-91 °C. ^1^H NMR (500 MHz, CDCl_3_): δ 7.88 (d, *J* = 9.0 Hz, 1H), 7.64 (d, *J* = 8.5 Hz, 2H), 7.35–7.31 (m, 1H), 7.29 (m, 2H), 7.21 (m, 1H), 7.18 (d, *J* = 9.0 Hz, 2H), 7.08 (d, *J* = 8.5 Hz, 2H), 6.99 (d, *J* = 8.5 Hz, 1H), 6.84 (d, *J* = 9.0 Hz, 1H), 3.98 (s, 3H, OMe), 3.80 (s, 3H, OMe), 1.32 (s, 12H, Bpin) ppm; ^13^C NMR (125 MHz, CDCl_3_): δ 156.83, 150.88, 148.36, 146.38, 144.54, 142.25, 142.17, 140.29, 136.09, 130.73, 130.71, 127.90, 125.36, 122.91, 122.88, 121.24, 121.12, 121.03, 120.93, 120.89, 119.88, 116.89, 115.10, 83.76, 58.12, 55.69, 25.07 ppm; ^19^F NMR (470 MHz, CDCl_3_): δ –146.81 (d, J = 9.4 Hz, 1F) ppm. HRMS (FAB) m/z: [M]^+^ calcd for C_30_H_31_BFNO_4_, 499.2330; found, 499.2326.

*Synthesis of* ***NPH****:* A heterogeneous mixture of anhydrous Toluene (12 mL), **3** (0.5 g, 0.73 mmol), **16** (1.48 g, 3.07 mmol), Pd(PPh_3_)_4_ (0.1 g, 12 mol %), K_2_CO_3_ (1.01 g, 10 eq),ethanol (8 ml) and DI water (8 ml) under argon was stirred under reflux for 12 h. The mixture was cooled and extracted with CH_2_Cl_2_. The organic layer was separated and dried over anhydrous MgSO_4_. Evaporation of the solvent gave a crude product, which was purified by silica gel column chromatography using CH_2_Cl_2_/hexane (3/1) as eluent to afford the desired product as a red solid in 82% yield. mp 226-227 °C. ^1^H NMR (500 MHz, CDCl_3_): δ 7.64 (d, *J* = 8.5 Hz, 1H), 7.62–7.58 (m, 3H), 7.52 (d, *J* = 8.5 Hz, 2H), 7.50–7.44 (m, 3H), 7.42 (d, *J* = 8.5 Hz, 2H), 7.40 (d, *J* = 8.5 Hz, 1H), 7.33–7.31 (m, 7H), 7.26–7.24 (m, 2H), 7.22–7.20 (m, 3H), 7.19–7.18 (m, 2H), 7.12–7.04 (m, 15H), 7.02–7.00 (m, 2H), 6.95–6.93 (m, 4H), 6.91–6.89 (m, 2H), 6.87–6.85 (m, 4H), 6.83–6.81 (m, 6H), 6.47 (s, 1H), 3.89 (s, 3H, OMe), 3.88 (s, 9H, OMe), 3.80 (s, 3H, OMe), 3.79 (s, 9H, OMe) ppm; ^13^C NMR (125 MHz, CDCl_3_): δ 189.29, 157.25, 157.21, 157.08, 156.69, 156.61, 156.41, 152.34, 148.74, 148.64, 147.83, 146.25, 145.73, 145.31, 143.62, 143.30, 141.93, 141.59, 140.69, 140.41, 138.29, 133.75, 131.48, 131.39, 131.20, 129.95, 128.90, 128.73, 128.68, 128.65, 128.07, 128.05, 127.99, 127.91, 127.60, 127.44, 127.27, 126.76, 126.69, 126.39, 126.23, 126.09, 125.81, 125.28, 125.13, 125.07, 122.30, 121.63, 121.39, 121.14, 120.69, 119.27, 119.16, 116.45, 115.12, 115.04, 115.00, 106.09, 55.69, 55.55 ppm; HRMS (MALDI) m/z: [M]^+^ calcd for C_114_H_84_N_4_O_9_S_4_, 1780.5121; found, 1780.5109.

*Synthesis of* ***NPF****:* A heterogeneous mixture of anhydrous Toluene (12 mL), **3** (0.5 g, 0.73 mmol), **17** (1.54 g, 3.08 mmol), Pd(PPh_3_)_4_ (0.1 g, 12 mol %), K_2_CO_3_ (1.01 g, 10 eq),ethanol (8 ml) and DI water (8 ml) under argon was stirred under reflux for 12 h. The mixture was cooled and extracted with CH_2_Cl_2_. The organic layer was separated and dried over anhydrous MgSO_4_. Evaporation of the solvent gave a crude product, which was purified by silica gel column chromatography using CH_2_Cl_2_/hexane (3/1) as eluent to afford the desired product as a red solid in 86% yield. mp 228-229 °C. ^1^H NMR (500 MHz, CDCl_3_): δ 7.65 (d, *J* = 9.0 Hz, 1H), 7.61 (d, *J* = 8.5 Hz, 3H), 7.53 (d, *J* = 4.0 Hz, 2H), 7.51–7.48 (m, 3H), 7.47–7.44 (m, 2H), 7.40 (d, *J* = 1.5 Hz, 1H), 7.34–7.24 (m, 7H), 7.22 (d, *J* = 2.0 Hz, 2H), 7.20–7.14 (m, 4H), 7.11–7.02 (m, 9H), 6.95 (d, *J* = 8.5 Hz, 2H), 6.94–6.89 (m, 4H), 6.89–6.86 (m, 3H), 6.86–6.80 (m, 12H), 6.49 (s, 1H), 3.90 (s, 3H, OMe), 3.88 (s, 9H, OMe), 3.87 (s, 3H, OMe), 3.86 (s, 9H, OMe) ppm; ^13^C NMR (125 MHz, CDCl_3_): δ 189.17, 157.46, 157.41, 157.30, 153.86, 151.90, 148.21, 148.12, 147.30, 145.80, 145.18, 144.52, 144.43, 144.34, 144.19, 142.79, 142.75, 141.89, 141.49, 140.88, 140.85, 140.82, 133.79, 131.77, 131.69, 131.53, 129.89, 129.84, 128.79, 128.72, 128.30, 128.21, 128.13, 126.85, 126.78, 126.59, 126.49, 126.31, 125.36, 125.21, 122.96, 122.35, 122.01, 121.84, 121.79, 121.41, 121.09, 120.94, 120.69, 120.48, 119.42, 119.39, 119.31, 114.37, 113.96, 113.80, 113.65, 106.08, 56.83, 55.55 ppm; ^19^F NMR (470 MHz, CDCl_3_): δ –132.51 to –132.75 (m, 4F) ppm; HRMS (FAB) m/z [M]^+^ calcd for C_114_H_80_F_4_N_4_O_9_S_4_: 1852.4744; found: 1852.4748.

*Synthesis of* ***NFP****:* A heterogeneous mixture of anhydrous Toluene (12 mL), **3** (0.5 g, 0.73 mmol), **18** (1.54 g, 3.08 mmol), Pd(PPh_3_)_4_ (0.1 g, 12 mol %), K_2_CO_3_ (1.01 g, 10 eq),ethanol (8 ml) and DI water (8 ml) under argon was stirred under reflux for 12 h. The mixture was cooled and extracted with CH_2_Cl_2_. The organic layer was separated and dried over anhydrous MgSO_4_. Evaporation of the solvent gave a crude product, which was purified by silica gel column chromatography using CH_2_Cl_2_/hexane (3/1) as eluent to afford the desired product as a red solid in 98% yield. mp 213-214 °C. **NFP:** ^1^H NMR (500 MHz, CDCl_3_): δ 7.91 (d, *J* = 9.5 Hz, 1H), 7.86 (d, *J* = 8.5 Hz, 3H), 7.53–7.45 (m, 5H), 7.37–7.28 (m, 10H), 7.21–7.12 (m, 8H), 7.07–7.02 (m, 10H), 6.96–6.92 (m, 4H), 6.90–6.84 (m, 8H), 6.81–6.79 (m, 7H), 3.98 (s, 3H, OMe), 3.97 (s, 9H, OMe), 3.81 (s, 3H, OMe), 3.79 (s, 9H, OMe) ppm; ^13^C NMR (125 MHz, CDCl_3_): δ 188.03, 187.39, 156.97, 156.94, 156.76, 151.82, 150.06, 149.44, 148.72, 148.62, 148.52, 148.31, 147.77, 147.04, 146.34, 144.53, 144.30, 144.23, 144.18, 142.33, 142.26, 141.73, 140.72, 140.23, 140.08, 140.05, 140.00, 139.95, 139.71, 137.51, 132.57, 130.68, 130.61, 128.92, 128.08, 127.84, 127.71, 127.58, 126.86, 126.78, 126.41, 126.30, 126.08, 125.33, 125.14, 125.03, 124.93, 124.47, 124.34, 122.89, 122.85, 122.72, 122.20, 122.10, 121.47, 121.25, 121.18, 121.04, 120.95, 120.23, 119.82, 119.73, 119.66, 119.19, 116.92, 116.33, 115.23, 115.17, 115.13, 113.20, 112.53, 110.65, 58.06, 55.69 ppm; ^19^F NMR (470 MHz, CDCl_3_): δ –146.73 (m, 4F) ppm. HRMS (MALDI) m/z: [M]^+^ calcd for C_114_H_80_F_4_N_4_O_9_S_4_, 1852.4744; found, 1852.4715.

**Figure S1.** ^1^H NMR spectrum of compound **10**.

**Figure S2.** ^13^C NMR spectrum of compound **10**.

**Figure S3.** MASS spectra of compound **10**.

**Figure S4.** ^1^H NMR spectrum of compound **11**.

**Figure S5.** ^13^C NMR spectrum of compound **11**.

**Figure S6.** ^19^F NMR spectrum of compound **11**.

**Figure S7.** MASS spectra of compound **11**.

**Figure S8.** ^1^H NMR spectrum of compound **12**.

**Figure S9.** ^13^C NMR spectrum of compound **12**.

**Figure S10.** ^19^F NMR spectrum of compound **12**.

**Figure S11.** MASS spectra of compound **12**.

**Figure S12.** ^1^H NMR spectrum of compound **13**.

**Figure S13.** ^13^C NMR spectrum of compound **13**.

**Figure S14.** MASS spectra of compound **13**.

**Figure S15.** ^1^H NMR spectrum of compound **14**.

**Figure S16.** ^13^C NMR spectrum of compound **14**.

**Figure S17.** ^19^F NMR spectrum of compound **14**.

**Figure S18.** MASS spectra of compound **14**.

**Figure S19.** ^1^H NMR spectrum of compound **15**.

**Figure S20.** ^13^C NMR spectrum of compound **15**.

**Figure S21.** ^19^F NMR spectrum of compound **15**.

**Figure S22.** MASS spectra of compound **15**.

**Figure S23.** ^1^H NMR spectrum of compound **16**.

**Figure S24.** ^13^C NMR spectrum of compound **16**.

**Figure S25.** MASS spectra of compound **16**.

**Figure S26.** ^1^H NMR spectrum of compound **17**.

**Figure S27.** ^13^C NMR spectrum of compound **17**.

**Figure S28.** ^19^F NMR spectrum of compound **17**.

**Figure S29.** MASS spectra of compound **17**.

**Figure S30.** ^1^H NMR spectrum of compound **18**.

**Figure S31.** ^13^C NMR spectrum of compound **18**.

**Figure S32.** ^19^F NMR spectrum of compound **18**.

**Figure S33.** MASS spectra of compound **18**.

**Figure S34.** ^1^H NMR spectrum of **NPH**.

**Figure S35.** ^13^C NMR spectrum of **NPH**.

**Figure S36.** MASS spectra of **NPH**.

**Figure S37.** ^1^H NMR spectrum of **NPF**.

**Figure S38.** ^13^C NMR spectrum of **NPF**.

**Figure S39.** ^19^F NMR spectrum of **NPF**.

**Figure S40.** MASS spectra of **NPF**.

**Figure S41.** ^1^H NMR spectrum of **NFP**.

**Figure S42.** ^13^C NMR spectrum of **NFP**.

**Figure S43.** ^19^F NMR spectrum of **NFP**.

**Figure S44.** MASS spectra of **NFP**.

**Figure S45.** The X-ray crystal structures of compound **3**.

**Figure S46.** TGA curves of the **NPH**, **NPF**, and **NFP**.

**Figure S47.** DSC curves of the (a) **NPH**, (b) **NPF**, and (c) **NFP**.

**Figure S48.** PXRD powder patterns of (a) **NPH**, (b) **NPF**, and (c) **NFP.**

**Figure S49**. Calculated absorption spectra of **NPH**, **NPF**, and **NFP** in THF solution.

**Figure S50**. Cyclic voltammograms of **NPH**, **NPF**, and **NFP** with 0.1 M TBAPF_6_ in THF at a scan rate of 100 mV^s−1^.

**Figure S51.** Space-charge-limited-current plots in the *J*-*V* characteristics of the devices with **NPH**, **NPF**, **NFP**, and spiro-OMeTAD as HTM, respectively.

**Figure S52.** XPS signals of Pb 4f, I 3d, and S 2p from a pristine **NPH** film and a **NPH** coated perovskite film

**Figure S53.** XPS signals of Pb 4f, I 3d, F 1s and S 2p from a pristine **NPF** film and a **NPF** coated perovskite film.

**Figure S54.** Schematic illustration of a fully blade-coating–based fabrication process combined with vacuum-assisted crystallization for n–i–p perovskite solar cells.

**Figure S55.** Plots of *J-V* curves for the perovskite solar cells using **NPH**, **NPF**, **NFP**, and spiro-OMeTAD as HTM under forward scan (at illumination of 100 mW/cm^2^, AM1.5G).

**Figure S56.** Plots of *J-V* curves for the perovskite solar cells using **NPH**, **NPF**, **NFP**, and spiro-OMeTAD as HTM without dopants under (a) reverse scan and (b) forward scan (at illumination of 100 mW/cm^2^, AM1.5G).

**Figure S57**. PCE vs. aging/oxidation time for **NPH**-, **NPF**-, **NFP**-, and spiro-OMeTAD-based perovskite solar devices.

**Figure S58.** The water contact angles on **NPH**, **NPF**, **NFP**, and spiro-OMeTAD films.

**Table S1** Summary of the fitting results and corresponding dynamic parameters derived from TRPL decay traces.

| HTM | A_1_ (%) | τ_1_ (ns) | A_2_ (%) | τ_2_ (ns) | τ_avg._ (ns) |
| --- | --- | --- | --- | --- | --- |
| PVSK | 26.57 | 4.60 | 73.42 | 35.30 | 27.14 |
| **NPH** | 45.96 | 4.05 | 54.03 | 21.95 | 13.72 |
| **NPF** | 45.29 | 2.14 | 54.70 | 15.19 | 9.28 |
| **NFP** | 47.81 | 1.43 | 52.18 | 11.97 | 6.93 |
| Spiro-OMeTAD | 46.16 | 2.38 | 53.84 | 15.92 | 9.67 |

**Table S2.** Photovoltaic parameters extracted from *J-V* measurements of PSCs based on the various HTMs without dopants.

| HTM | Scan direction | *V*_oc_ [V] | *J*_sc_ [mA cm^-2^] | FF (%) | PCE_max_ [%] |
| --- | --- | --- | --- | --- | --- |
| **NPH** | Reverse | 0.98 | 18.28 | 65 | 11.64 |
|  | Forward | 0.96 | 18.30 | 58 | 10.19 |
| **NPF** | Reverse | 1.01 | 21.36 | 63 | 13.59 |
|  | Forward | 1.01 | 21.45 | 60 | 13.00 |
| **NFP** | Reverse | 1.00 | 22.11 | 68 | 15.03 |
|  | Forward | 0.97 | 22.43 | 58 | 12.62 |
| **Spiro-OMeTAD** | Reverse | 1.03 | 19.34 | 66 | 13.15 |
|  | Forward | 1.01 | 19.54 | 52 | 10.26 |

**Table S3.** Comparison between the previously reported spiro-CPDT HTMs and the HTMs developed in the present work.

| **Parameter** | **spiro-1** | **spiro-2** | **NPH** | **NPF** | **NFP** |
| --- | --- | --- | --- | --- | --- |
| Reference | Chem. Eng. J. 2023, 454, 139926 | Chem. Eng. J. 2023, 454, 139926 | This work | This work | This work |
| Central core | Spiro-CPDT/benzodithiophenone-derived core | Spiro-CPDT/benzodithiophenone-derived core | Same spiro-CPDT core platform | Same spiro-CPDT core platform | Same spiro-CPDT core platform |
| Peripheral structure | Four symmetric phenyl-linked triarylamine donor arms | Four symmetric thiophene-extended triarylamine donor arms | Four asymmetric phenylnaphthylamine arms | Fluorinated asymmetric phenylnaphthylamine arms | Regioisomeric fluorinated asymmetric phenylnaphthylamine arms |
| Principal structural variable | Phenylene linker | Thiophene-extended linker | Non-fluorinated asymmetric reference | Fluorination at one terminal position | Fluorination at an alternative terminal position |
| Molecular-design purpose | D–A–D spiro HTM with facile synthesis | Extended conjugation through thiophene linkers | Extend π-conjugation through naphthyl groups | Evaluate fluorination | Evaluate fluorine-position effect |
| Spiro-core formation | Previously reported one-pot reaction; crystallization purification | Same | Uses the previously reported core-forming platform | Same | Same |
| Number of isolated synthetic steps from selected starting materials¹ | 2 | 2 | 6 | 6 | 6 |
| Spiro-core intermediate yield | 50% | 50% | 31% over bromination and spiro-forming steps² | 31% | 31% |
| Final coupling yield | 84% | 78% | 82% | 86% | 98% |
| Overall isolated yield, longest linear sequence³ | 42% | 39% | ≈27% | ≈29% | ≈41% |
| Final purification | Silica-gel chromatography | Silica-gel chromatography | Silica-gel chromatography | Silica-gel chromatography | Silica-gel chromatography |
| Device architecture | FTO/c-TiO₂/mp-TiO₂/perovskite/HTM/Ag | Same | ITO/SnO₂/perovskite/HTM/Ag | Same | Same |
| Functional-layer deposition | Spin coating; mp-TiO₂ screen printing | Same | Blade-coated SnO₂, perovskite, and HTM layers; thermally evaporated Ag | Same | Same |
| Crystallization process | Antisolvent-assisted spin coating | Same | Vacuum-assisted crystallization | Same | Same |
| Small-device active area | 0.16 cm² | 0.16 cm² | 0.09 cm² | 0.09 cm² | 0.09 cm² |
| Best small-area PCE | 21.67% | 19.65% | 22.28% | 23.83% | 24.49% |
| Average PCE | 21.12% | 19.26% | 22.14% | 23.72% | 24.41% |
| Large-area result | Not reported | Not reported | Not emphasized | Not emphasized | 23.28% at 1.00 cm² |
| Dark-storage stability | >85% retained after 1000 h at 20–25 °C and 30–40% RH, unencapsulated | >85% retained after 1000 h | 86% after 1000 h at ≤40% RH, unencapsulated | 89% after 1000 h | 92% after 1000 h |
| Thermal stability | ~80% retained after 100 h at 85 °C in air | ~80% retained after 100 h | 78% after 400 h at 85 °C | 85% after 400 h | 88% after 400 h |
| Continuous-illumination stability | >80% retained after 500 h under 1 sun at 50 °C | >80% retained after 500 h | 76% after 400 h under 1 sun at 40 °C in N₂ | >80% after 400 h | >80% after 400 h |

^1^ The step count includes isolated transformations needed to prepare both the spiro-core intermediate and the terminal coupling precursor, followed by the final Suzuki coupling. Because **NPH**, **NPF**, and **NFP** are prepared through convergent synthetic routes, the numerical step count should not be interpreted as a single uninterrupted linear sequence.

^2^ The spiro-core precursor used in the present work was obtained through bromination in 62% yield followed by spiro-core formation in 50% yield, corresponding to approximately 31% over the two steps.

^3^ For the convergent syntheses of **NPH**, **NPF**, and **NFP**, the values represent the product of the isolated yields along the longest terminal-unit sequence, including the final coupling step. They are provided for a consistent route-level comparison and do not represent a mass-balance-based process yield incorporating both convergent branches.

**Table S4**. Cost calculation for Compound **2**.

| **Reagent** | **Amount (g)** | **Amount (mL)** | **Unit price (US$/g or US$/mL)** | **Line cost (US$)** |
| --- | --- | --- | --- | --- |
| 4H-Cyclopenta[1,2-b:5,4-b′]dithiophen-4-one (Compound 1) | 1 |  | 20 | 20 |
| N-Bromosuccinimide (NBS) | 2.32 |  | 0.0940 | 0.2181 |
| Tetrahydrofuran (THF) |  | 20 | 0.0235 | 0.4700 |
| Hexane |  | 700 | 0.0011 | 0.7700 |
| Silica gel | 40 |  | 0.0042 | 0.1680 |
| Total material cost | | | | US$21.626 |
| Calculated product amount (SI yield) | | | | 1.129 g (62%) |
| **Material cost of Compound 2** | | | | **US$19.158/g** |

**Table S5**. Cost calculation for Compound **3**.

| **Reagent** | **Amount (g)** | **Amount (mL)** | **Unit price (US$/g or US$/mL)** | **Line cost (US$)** |
| --- | --- | --- | --- | --- |
| Compound **2** | 1 |  | 19.1585 | 19.1585 |
| Triisopropyl phosphite, P(O-iPr)₃ |  | 1.54 | 0.6340 | 0.9765 |
| Dichloromethane |  | 20 | 0.0046 | 0.0920 |
| Total material cost | | | | US$20.227 |
| Calculated product amount (SI yield) | | | | 0.489 g (50%) |
| **Material cost of Compound 3** | | | | **US$41.398/g** |

**Table S6**. Cost calculation for Compound **10**.

| **Reagent** | **Amount (g)** | **Amount (mL)** | **Unit price (US$/g or US$/mL)** | **Line cost (US$)** |
| --- | --- | --- | --- | --- |
| 2-Bromo-6-methoxynaphthalene (Compound **4**) | 1 |  | 1.4 | 1.4 |
| p-Anisidine (Compound **5**) | 0.4 |  | 0.3500 | 0.1400 |
| Pd₂(dba)₃ | 0.04 |  | 20.2 | 0.8080 |
| P(t-Bu)₃·HBF₄ | 0.04 |  | 41.199 | 1.648 |
| Sodium tert-butoxide (NaOtBu) | 0.4 |  | 0.6580 | 0.2632 |
| Toluene |  | 12 | 0.0040 | 0.0480 |
| Ethyl acetate |  | 100 | 0.0033 | 0.3300 |
| Hexane |  | 700 | 0.0011 | 0.7700 |
| Silica gel | 40 |  | 0.0042 | 0.1680 |
| Total material cost | | | | US$5.575 |
| Calculated product amount (SI yield) | | | | 0.744 g (82%) |
| **Material cost of Compound 10** | | | | **US$7.493/g** |

**Table S7**. Cost calculation for Compound **13**.

| **Reagent** | **Amount (g)** | **Amount (mL)** | **Unit price (US$/g or US$/mL)** | **Line cost (US$)** |
| --- | --- | --- | --- | --- |
| Compound **10** | 1 |  | 7.4935 | 7.4935 |
| 1-Bromo-4-iodobenzene | 1.4 |  | 3.453 | 4.8342 |
| Pd₂(dba)₃ | 0.1 |  | 20.2 | 2.02 |
| DPPF | 0.14 |  | 0.8000 | 0.1120 |
| Sodium tert-butoxide (NaOtBu) | 0.6 |  | 0.6580 | 0.3948 |
| Toluene |  | 12 | 0.0040 | 0.0480 |
| Ethyl acetate |  | 75 | 0.0033 | 0.2475 |
| Hexane |  | 700 | 0.0011 | 0.7700 |
| Silica gel | 40 |  | 0.0042 | 0.1680 |
| Total material cost | | | | US$16.088 |
| Calculated product amount (SI yield) | | | | 1.042 g (67%) |
| **Material cost of Compound 13** | | | | **US$15.442/g** |

**Table S8**. Cost calculation for Compound **16**.

| **Reagent** | **Amount (g)** | **Amount (mL)** | **Unit price (US$/g or US$/mL)** | **Line cost (US$)** |
| --- | --- | --- | --- | --- |
| Compound **13** | 1 |  | 15.4425 | 15.4425 |
| Bis(pinacolato)diboron (B₂pin₂) | 0.7 |  | 0.5600 | 0.3920 |
| Pd(PPh₃)₂Cl₂ | 0.04 |  | 20.36 | 0.8144 |
| Potassium acetate (KOAc) | 0.02 |  | 0.0250 | 0.0005 |
| Toluene |  | 24 | 0.0040 | 0.0960 |
| Ethyl acetate |  | 150 | 0.0033 | 0.4950 |
| Hexane |  | 700 | 0.0011 | 0.7700 |
| Silica gel | 40 |  | 0.0042 | 0.1680 |
| Total material cost | | | | US$18.178 |
| Calculated product amount (SI yield) | | | | 0.665 g (60%) |
| **Material cost of Compound 16** | | | | **US$27.336/g** |

Table S9. Cost calculation for **NPH**.

| **Reagent** | **Amount (g)** | **Amount (mL)** | **Unit price (US$/g or US$/mL)** | **Line cost (US$)** |
| --- | --- | --- | --- | --- |
| Compound **3** | 1 |  | 41.3979 | 41.3979 |
| Compound **16** | 2.96 |  | 27.3359 | 80.9144 |
| Pd(PPh₃)₄ | 0.2 |  | 22.24 | 4.448 |
| K₂CO₃ | 2.02 |  | 0.0300 | 0.0606 |
| Water |  | 16 | 0.0000 | 0.0000 |
| Ethanol |  | 16 | 0.0055 | 0.0880 |
| Toluene |  | 24 | 0.0040 | 0.0960 |
| Dichloromethane |  | 400 | 0.0046 | 1.84 |
| Hexane |  | 700 | 0.0011 | 0.7700 |
| Silica gel | 40 |  | 0.0042 | 0.1680 |
| Total material cost | | | | US$129.783 |
| Calculated product amount (SI yield) | | | | 2.136 g (82%) |
| **Material cost of NPH** | | | | **US$60.754/g** |

**Table S10**. Cost calculation for Compound **11**.

| **Reagent** | **Amount (g)** | **Amount (mL)** | **Unit price (US$/g or US$/mL)** | **Line cost (US$)** |
| --- | --- | --- | --- | --- |
| 2-Bromo-6-methoxynaphthalene (Compound **6**) | 1 |  | 1.4 | 1.4 |
| 3-Fluoro-4-methoxyaniline (Compound **7**) | 0.6 |  | 0.6000 | 0.3600 |
| Pd₂(dba)₃ | 0.06 |  | 20.2 | 1.212 |
| P(t-Bu)₃·HBF₄ | 0.04 |  | 41.199 | 1.648 |
| Sodium tert-butoxide (NaOtBu) | 0.8 |  | 0.6580 | 0.5264 |
| Toluene |  | 12 | 0.0040 | 0.0480 |
| Ethyl acetate |  | 100 | 0.0033 | 0.3300 |
| Hexane |  | 700 | 0.0011 | 0.7700 |
| Silica gel | 40 |  | 0.0042 | 0.1680 |
| Total material cost | | | | US$6.462 |
| Calculated product amount (SI yield) | | | | 0.778 g (62%) |
| **Material cost of Compound 11** | | | | **US$8.311/g** |

**Table S11**. Cost calculation for Compound **14**.

| **Reagent** | **Amount (g)** | **Amount (mL)** | **Unit price (US$/g or US$/mL)** | **Line cost (US$)** |
| --- | --- | --- | --- | --- |
| Compound **11** | 1 |  | 8.3106 | 8.3106 |
| 1-Bromo-4-iodobenzene | 1.4 |  | 3.453 | 4.8342 |
| Pd₂(dba)₃ | 0.1 |  | 20.2 | 2.02 |
| DPPF | 0.14 |  | 0.8000 | 0.1120 |
| Sodium tert-butoxide (NaOtBu) | 0.6 |  | 0.6580 | 0.3948 |
| Toluene |  | 12 | 0.0040 | 0.0480 |
| Ethyl acetate |  | 75 | 0.0033 | 0.2475 |
| Hexane |  | 700 | 0.0011 | 0.7700 |
| Silica gel | 40 |  | 0.0042 | 0.1680 |
| Total material cost | | | | US$16.905 |
| Calculated product amount (SI yield) | | | | 1.050 g (69%) |
| **Material cost of Compound 14** | | | | **US$16.106/g** |

**Table S12**. Cost calculation for Compound **17**.

| **Reagent** | **Amount (g)** | **Amount (mL)** | **Unit price (US$/g or US$/mL)** | **Line cost (US$)** |
| --- | --- | --- | --- | --- |
| Compound **14** | 1 |  | 16.1063 | 16.1063 |
| Bis(pinacolato)diboron (B₂pin₂) | 0.68 |  | 0.5600 | 0.3808 |
| Pd(PPh₃)₂Cl₂ | 0.04 |  | 20.36 | 0.8144 |
| Potassium acetate (KOAc) | 0.02 |  | 0.0250 | 0.0005 |
| Toluene |  | 24 | 0.0040 | 0.0960 |
| Ethyl acetate |  | 150 | 0.0033 | 0.4950 |
| Hexane |  | 700 | 0.0011 | 0.7700 |
| Silica gel | 40 |  | 0.0042 | 0.1680 |
| Total material cost | | | | US$18.831 |
| Calculated product amount (SI yield) | | | | 0.883 g (80%) |
| **Material cost of Compound 17** | | | | **US$21.321/g** |

**Table S13**. Cost calculation for **NPF**.

| **Reagent** | **Amount (g)** | **Amount (mL)** | **Unit price (US$/g or US$/mL)** | **Line cost (US$)** |
| --- | --- | --- | --- | --- |
| Compound **3** | 1 |  | 41.3979 | 41.3979 |
| Compound **17** | 3.08 |  | 21.3213 | 65.6696 |
| Pd(PPh₃)₄ | 0.2 |  | 22.24 | 4.448 |
| K₂CO₃ | 2.02 |  | 0.0300 | 0.0606 |
| Water |  | 16 | 0.0000 | 0.0000 |
| Ethanol |  | 16 | 0.0055 | 0.0880 |
| Toluene |  | 24 | 0.0040 | 0.0960 |
| Dichloromethane |  | 400 | 0.0046 | 1.84 |
| Hexane |  | 700 | 0.0011 | 0.7700 |
| Silica gel | 40 |  | 0.0042 | 0.1680 |
| Total material cost | | | | US$114.538 |
| Calculated product amount (SI yield) | | | | 2.331 g (86%) |
| **Material cost of NPF** | | | | **US$49.137/g** |

**Table S14**. Cost calculation for Compound **12**.

| **Reagent** | **Amount (g)** | **Amount (mL)** | **Unit price (US$/g or US$/mL)** | **Line cost (US$)** |
| --- | --- | --- | --- | --- |
| 6-Bromo-1-fluoro-2-methoxynaphthalene (Compound **8**) | 1 |  | 600.000 | 600.000 |
| p-Anisidine (Compound **9**) | 0.6 |  | 0.3500 | 0.2100 |
| Pd₂(dba)₃ | 0.08 |  | 20.2 | 1.616 |
| P(t-Bu)₃·HBF₄ | 0.04 |  | 41.199 | 1.648 |
| Sodium tert-butoxide (NaOtBu) | 0.76 |  | 0.6580 | 0.5001 |
| Toluene |  | 12 | 0.0040 | 0.0480 |
| Ethyl acetate |  | 100 | 0.0033 | 0.3300 |
| Hexane |  | 700 | 0.0011 | 0.7700 |
| Silica gel | 40 |  | 0.0042 | 0.1680 |
| Total material cost | | | | US$605.290 |
| Calculated product amount (SI yield) | | | | 1.002 g (86%) |
| **Material cost of Compound 12** | | | | **US$603.841/g** |

**Table S15**. Cost calculation for Compound **15**.

| **Reagent** | **Amount (g)** | **Amount (mL)** | **Unit price (US$/g or US$/mL)** | **Line cost (US$)** |
| --- | --- | --- | --- | --- |
| Compound **12** | 1 |  | 603.841 | 603.841 |
| 1-Bromo-4-iodobenzene | 1.4 |  | 3.453 | 4.8342 |
| Pd₂(dba)₃ | 0.1 |  | 20.2 | 2.02 |
| DPPF | 0.14 |  | 0.8000 | 0.1120 |
| Sodium tert-butoxide (NaOtBu) | 0.6 |  | 0.6580 | 0.3948 |
| Toluene |  | 12 | 0.0040 | 0.0480 |
| Ethyl acetate |  | 75 | 0.0033 | 0.2475 |
| Hexane |  | 700 | 0.0011 | 0.7700 |
| Silica gel | 40 |  | 0.0042 | 0.1680 |
| Total material cost | | | | US$612.435 |
| Calculated product amount (SI yield) | | | | 1.232 g (81%) |
| **Material cost of Compound 15** | | | | **US$497.026/g** |

**Table S16**. Cost calculation for Compound **18**.

| **Reagent** | **Amount (g)** | **Amount (mL)** | **Unit price (US$/g or US$/mL)** | **Line cost (US$)** |
| --- | --- | --- | --- | --- |
| Compound **15** | 1 |  | 497.026 | 497.026 |
| Bis(pinacolato)diboron (B₂pin₂) | 0.68 |  | 0.5600 | 0.3808 |
| Pd(PPh₃)₂Cl₂ | 0.04 |  | 20.36 | 0.8144 |
| Potassium acetate (KOAc) | 0.02 |  | 0.0250 | 0.0005 |
| Toluene |  | 24 | 0.0040 | 0.0960 |
| Ethyl acetate |  | 150 | 0.0033 | 0.4950 |
| Hexane |  | 700 | 0.0011 | 0.7700 |
| Silica gel | 40 |  | 0.0042 | 0.1680 |
| Total material cost | | | | US$499.751 |
| Calculated product amount (SI yield) | | | | 0.662 g (60%) |
| **Material cost of Compound 18** | | | | **US$754.454/g** |

**Table S17**. Cost calculation for **NFP**.

| **Reagent** | **Amount (g)** | **Amount (mL)** | **Unit price (US$/g or US$/mL)** | **Line cost (US$)** |
| --- | --- | --- | --- | --- |
| Compound **3** | 1 |  | 41.3979 | 41.3979 |
| Compound **18** | 3.08 |  | 754.454 | 2323.720 |
| Pd(PPh₃)₄ | 0.2 |  | 22.24 | 4.448 |
| K₂CO₃ | 2.02 |  | 0.0300 | 0.0606 |
| Water |  | 16 | 0.0000 | 0.0000 |
| Ethanol |  | 16 | 0.0055 | 0.0880 |
| Toluene |  | 24 | 0.0040 | 0.0960 |
| Dichloromethane |  | 400 | 0.0046 | 1.84 |
| Hexane |  | 700 | 0.0011 | 0.7700 |
| Silica gel | 40 |  | 0.0042 | 0.1680 |
| Total material cost | | | | US$2372.588 |
| Calculated product amount (SI yield) | | | | 2.656 g (98%) |
| **Material cost of NFP** | | | | **US$893.226/g** |

References

[1] H. R. Tseng, H. Phan, C. Luo, M. Wang, L. A. Perez, S. N. Patel, L. Ying, E. J. Kramer, T. Q. Nguyen, G. C. Bazan, A. J. Heeger, Adv. Mater. 2014, 26, 2993−2998.

[2] T. Yanai, D. Tew, and N. Handy, “A new hybrid exchange-correlation functional using the Coulomb-attenuating method (CAM-B3LYP),” Chem. Phys. Lett., 2004, 393, 51-57.

[3] Gaussian 16, Revision A.03, M. J. Frisch, G. W. Trucks, H. B. Schlegel, G. E. Scuseria, M. A. Robb, J. R. Cheeseman, G. Scalmani, V. Barone, G. A. Petersson, H. Nakatsuji, X. Li, M. Caricato, A. V. Marenich, J. Bloino, B. G. Janesko, R. Gomperts, B. Mennucci, H. P. Hratchian, J. V. Ortiz, A. F. Izmaylov, J. L. Sonnenberg, D. Williams-Young, F. Ding, F. Lipparini, F. Egidi, J. Goings, B. Peng, A. Petrone, T. Henderson, D. Ranasinghe, V. G. Zakrzewski, J. Gao, N. Rega, G. Zheng, W. Liang, M. Hada, M. Ehara, K. Toyota, R. Fukuda, J. Hasegawa, M. Ishida, T. Nakajima, Y. Honda, O. Kitao, H. Nakai, T. Vreven, K. Throssell, J. A. Montgomery, Jr., J. E. Peralta, F. Ogliaro, M. J. Bearpark, J. J. Heyd, E. N. Brothers, K. N. Kudin, V. N. Staroverov, T. A. Keith, R. Kobayashi, J. Normand, K. Raghavachari, A. P. Rendell, J. C. Burant, S. S. Iyengar, J. Tomasi, M. Cossi, J. M. Millam, M. Klene, C. Adamo, R. Cammi, J. W. Ochterski, R. L. Martin, K. Morokuma, O. Farkas, J. B. Foresman, and D. J. Fox, Gaussian, Inc., Wallingford CT, 2016.

[4] J. Tomasi, B. Mennucci, and R. Cammi, “Quantum mechanical continuum solvation models,” Chem. Rev., 2005, 105, 2999-3093.

[5] C. Chen, S. P. Ong, A universal graph deep learning interatomic potential for the periodic table, Nat. Comput. Sci. 2022, 2, 718–728.

[6] S. Grimme, S. Ehrlich, and L. Goerigk, Effect of the Damping Function in Dispersion Corrected Density Functional Theory, J. Comput. Chem., 2011, 32, 1456.

[7] S. Grimme, C. Bannwarth, P. Shushkov, A Robust and Accurate Tight-Binding Quantum Chemical Method for Structures, Vibrational Frequencies, and Noncovalent Interactions of Large Molecular Systems Parametrized for All spd-Block Elements (Z = 1-86), J. Chem. Theory Comput., 2017, 13 (5), pp 1989–2009

[8] AMS 2024.1, SCM, Theoretical Chemistry, Vrije Universiteit, Amsterdam, The Netherlands, <http://www.scm.com>.
